# Long noncoding RNA Lnc-DIF inhibits bone formation by sequestering miR-489-3p

**DOI:** 10.1016/j.isci.2022.103949

**Published:** 2022-02-21

**Authors:** Chong Yin, Ye Tian, Dijie Li, Yang Yu, Shanfeng Jiang, Yimei Hou, Meng Deng, Kaiyuan Zheng, Yan Zhang, Xiaoni Deng, Zhihao Chen, Zhiping Miao, Qiang Hao, Yu Li, Airong Qian

**Affiliations:** 1 Lab for Bone Metabolism, Xi'an Key Laboratory of Special Medicine and Health Engineering, Key Lab for Space Biosciences and Biotechnology, Research Center for Special Medicine and Health Systems Engineering, NPU-UAB Joint Laboratory for Bone Metabolism, School of Life Sciences, Northwestern Polytechnical University, 127 West Youyi Road, Xi'an, Shaanxi 710072, China; 2 Department of Clinical Laboratory, Academician (expert) Workstation, Lab of Epigenetics and RNA Therapy, Affiliated Hospital of North Sichuan Medical College, Nanchong, P.R.China; 3 Department of Laboratory Medicine, Translational Medicine Research Center, North Sichuan Medical College, Nanchong, P.R.China; 4 Tianjin Key Laboratory on Technologies Enabling Development Clinical Therapeutics and Diagnostics (Theranostics), School of Pharmacy, Tianjin Medical University, Tianjin, China; 5 State Key Laboratory of Cancer Biology, Biotechnology Center, School of Pharmacy, Fourth Military Medical University, Xi’an 710032, China

**Keywords:** Biological sciences, Molecular biology, Molecular mechanism of gene regulation, Orthopedics

## Abstract

Osteoporosis has become a high incident bone disease along with the aging of human population. Long noncoding RNAs (LncRNAs) play an important role in osteoporosis incidence. In this study, we screened out an LncRNA negatively correlated with osteoblast differentiation, which was therefore named Lnc-DIF (differentiation inhibiting factor). Functional analysis proved that Lnc-DIF inhibited bone formation. A special structure containing multiple 53 nucleotide repeats was found in the trailing end of Lnc-DIF. Our study suggested that this repeat sequence could sequester multiple miR-489-3p and inhibit bone formation through miR-489-3p/SMAD2 axis. Moreover, siRNA of Lnc-DIF would rescue bone formation in both aging and ovariectomized osteoporosis mice. This study revealed a kind of LncRNA that could function as a sponge and regulate multiple miRNAs. RNA therapy techniques that target these LncRNAs could manipulate its downstream miRNA-target pathway with significantly higher efficiency and specificity. This provided potential therapeutic insight for RNA-based therapy for osteoporosis.

## Highlights

• Identified LncRNA Lnc-DIF that inhibited bone formation
• Lnc-DIF sequestered multiple miR-489-3p by the repeat sequences on its trailing end
• Lnc-DIF repeat sequence inhibited bone formation via miR-489-3p/SMAD2 axis
• Lnc-DIF siRNA showed strong capability on rescuing osteoporosis

## Graphical abstract

**Figure:**
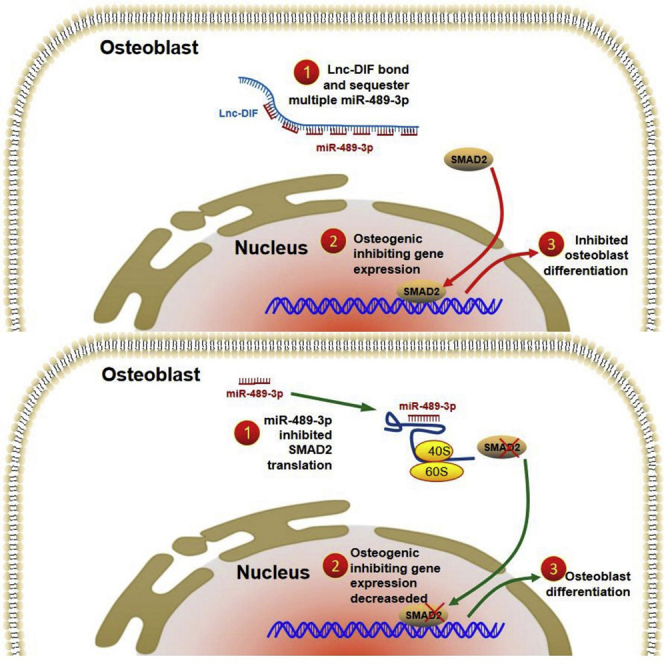

## Introduction

Osteoporosis has become an emerging threat to human health. Its incidence is elevating along with the aging of the population. Osteoporosis can be caused by several factors, including heredity, aging, and postmenopausal hormone disorder (Clarke and Khosla, 2010). Although impacted by multiple factors, the formation and development of osteoporosis is largely attributed to reduced bone formation that resulted from decreased osteoblast differentiation (Marie, 1999). The differentiation of osteoblast is a multistep process controlled by a variety of genetic and epigenetic signaling pathways. Long noncoding RNAs (LncRNAs) have been proved as an essential regulator to osteoblast differentiation among all epigenetic pathways (Hassan et al., 2015; Peng et al., 2018a, 2018b), and it glittered as a highlight in recent bone research.

LncRNA is a kind of transcript composed of more than 200 nucleotides and usually with no coding potential. LncRNAs have been reported to regulate multiple physiological and pathological processes including development, metabolism, cancer, and musculoskeletal system function (Batista and Chang, 2013; Geisler and Coller, 2013, Meng et al., 2015, Yan et al., 2016). Several mechanisms for LncRNA regulation have been characterized, including histone modification (Zhao et al., 2008), transcription factor regulation (Hung et al., 2011), alternative splicing (Tripathi et al., 2010), and competing endogenous RNA (ceRNA) of miRNAs (Salmena et al., 2011; Thomson and Dinger, 2016). By sponging miRNAs, the LncRNAs protect corresponding mRNA from being silenced. Researchers have identified several LncRNAs that regulated osteoblast differentiation by competing miRNA, including ODSM, H19, KCNQ1OT1, XIST, MALAT1, PGC1β-OT1, etc. (Feng et al., 2020; Li et al., 2020; Wang et al., 2018, 2019, 2020; Wu et al., 2018; Wu et al., 2019; Yi et al., 2019; Yuan et al., 2019). These findings provided essential theoretical basis for the significance of ceRNAs in manipulating bone formation. However, most competing LncRNAs could only sequester multiple different sorts of microRNAs at random binding sites, which resulted in low efficiency and low specificity. Studies about osteoblastic LncRNA that could interact with one target microRNA with multiple binding sites were relatively limited.

In this study, we discovered an LncRNA that inhibited osteoblast differentiation and bone formation, which was therefore named Lnc-DIF (differentiation inhibiting factor). A special region of thirteen repeats with 53nt length in the trailing end part of Lnc-DIF sequence was illustrated. Lnc-DIF could efficiently sequester miR-489-3p through its repeat sequences and further function as therapeutic target for osteoporosis. The study had discovered a kind of LncRNAs that contain repeat sequences. This special repeat sequence might sequester multiple miRNAs with higher specificity and efficiency. Manipulating the levels of these LncRNAs would evade the low homology of LncRNAs between different species and might provide more ideas to RNA-based therapeutic strategy of osteoporosis.

## Results

### Lnc-DIF was associated with the reduction of bone formation

**Figure 1:**
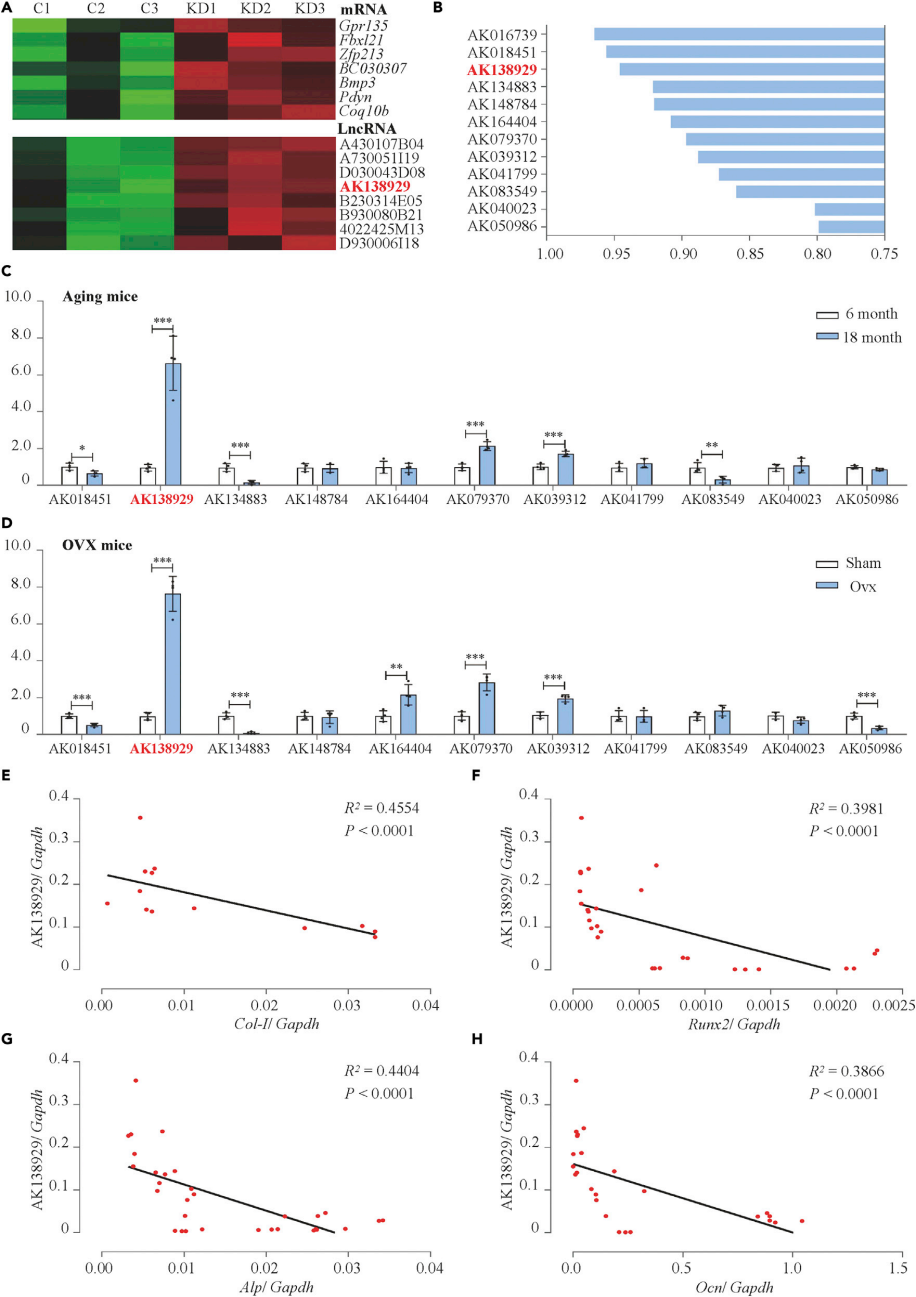
Identification of Lnc-DIF, which was associated with bone formation reduction (A) Selected area of mRNA array (up) and LncRNA array (bottom) heatmap for MACF1-knockdown MC3T3-E1 cell (KD1–KD3) and negative control (C1–C3). (B) Result of Gene Co-expression Networks analysis for LncRNA array and osteogenic mRNA array data. (C) Expression levels of LncRNA in BMSCs of 6- and 18-month-old male C57BL/6 mice, as detected by RT-PCR (mean ± SD, ∗∗p < 0.01, ∗∗∗p < 0.001, N = 3). (D) Expression levels of LncRNA in BMSCs of OVX C57BL/6 mice, as detected by RT-PCR (mean ± SD, ∗p < 0.05, ∗∗p < 0.01, ∗∗∗p < 0.001, N = 3). Sham: sham OVX operation group. OVX: OVX group. (E–H) Correlation analysis between Lnc-DIF levels and *Col Iα1*, *Runx2*, *Alp,* and *Ocn* mRNA levels in femur tissues from C57BL/6 mice, as detected by RT-PCR.

LncRNAs and mRNAs that were associated with osteoblast differentiation and bone formation were screened by microarray analysis using MACF1 (a cytoskeletal protein positively regulate osteoblast differentiation and bone formation via multiple osteogenic transcription factors) knockdown and control MC3T3-E1 cell lines as described previously (Yin et al., 2019, Figure 1A). To identity more osteogenic LncRNAs, co-expression network of LncRNAs and mRNAs that were associated with osteogenic signaling pathways (including Wnt, BMP, TGF-beta, and HIF-1 signaling pathway) was established by calculating the absolute value of average Pearson's correlation coefficient (PCC). Osteogenic LncRNA AK016739, which have been reported previously (Yin et al., 2019), presented most significant absolute co-expression level (0.96434). The other 11 LncRNAs with most significant absolute co-expression levels (>0.80) were selected as potential osteogenic-related LncRNAs (Figure 1B).

Next, the expression pattern of these 11 selected LncRNAs was investigated in bone marrow mesenchymal stem cells (BMSCs) isolated from aging and OVX osteoporosis mice. In 18-month-old aging mice, RT-PCR results showed that the expression level of AK138929 was the most increased one among all LncRNAs compared with 6-month control. The expression level of AK138929 was also the highest in OVX mice (Figures 1C and 1D).

The distribution of AK138929 in multiple tissues of C57BL/6 mice indicated that femur tissue had the highest expression level of AK138929 compared with all other organs including heart, liver, spleen, lung, kidney, and brain (Figure S1). This suggested that AK138929 may involve in osteogenesis.

In addition, the correlation analysis showed that there was a negative relationship between AK138929 expression and osteogenic marker genes *Col Iα1* (collagen type I), *Alp* (alkaline phosphatase), *Ocn* (osteocalcin), and *Runx2* (runt-related transcription factor 2) in the femur tissue of different ages of C57BL/6 mice (Figures 1E–1H). These data suggested that AK138929 might function as an osteogenic differentiation inhibiting factor, thus we named it as Lnc-DIF (differentiation inhibiting factor).

### Lnc-DIF inhibited osteoblast differentiation and bone formation

**Figure 2:**
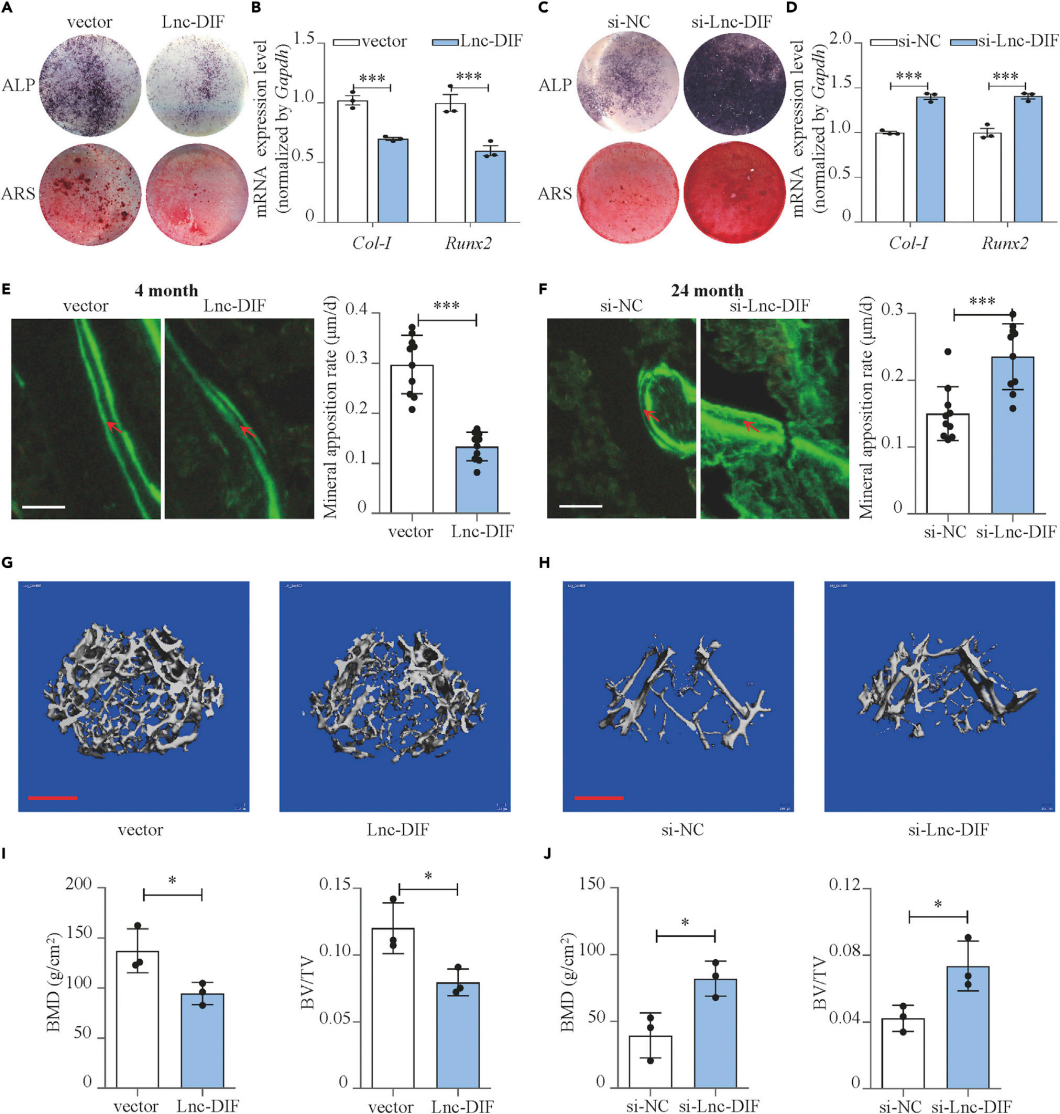
Lnc-DIF inhibited osteoblast differentiation and bone formation (A) Alp and Alizarin red staining of MC3T3-E1 cell treated with Lnc-DIF overexpression plasmid (compared with normal control), as detected by Alp staining and Alizarin red staining. Alp: results of Alp staining. Alz: results of Alizarin red staining. (B) *Col-I* and *Runx2* expression levels of MC3T3-E1 cell treated with Lnc-DIF overexpression plasmid, as detected by RT-PCR (mean ± SD, ∗∗∗p < 0.001, N = 3). (C) Alp and Alizarin red staining of MC3T3-E1 cell treated with siRNA-Lnc-DIF (compared with negative control siRNA), as detected by Alp staining and Alizarin red staining. Alp: results of Alp staining. Alz: results of Alizarin red staining. (D) *Col-I* and *Runx2* expression levels of MC3T3-E1 cell treated with siRNA-Lnc-DIF, as detected by RT-PCR (mean ± SD, ∗∗∗p < 0.001, N = 3). (E) Representative images showing femoral trabecular bone mineral apposition rate of C57BL/6 mice treated with Lnc-DIF overexpression plasmid (mean ± SD, ∗∗∗p < 0.001, N = 10). Scale bar: 10μm. (F) Representative images showing femoral trabecular bone mineral apposition rate of 24-month-old C57BL/6 mice treated with siRNA-Lnc-DIF (mean ± SD, ∗∗∗p < 0.001, N = 10). Scale bar: 10μm. (G) Representative images showing femoral trabecular microarchitecture of C57BL/6 mice treated with Lnc-DIF overexpression plasmid, as detected by micro CT. Scale bar: 500μm. (H) Representative images showing femoral trabecular microarchitecture of 24-month-old C57BL/6 mice treated with siRNA-Lnc-DIF, as detected by micro CT. Scale bar: 500μm. (I) Femoral Bone mineral density (BMD) and bone volume to tissue volume (BV/TV) of C57BL/6 mice treated with Lnc-DIF overexpression plasmid (mean ± SD, ∗p < 0.05, N = 3). (J) Femoral bone mineral density (BMD) and bone volume to tissue volume (BV/TV) of 24-month-old C57BL/6 mice treated with siRNA-Lnc-DIF (mean ± SD, ∗p < 0.05, N = 3).

To investigate the function of Lnc-DIF on osteoblast differentiation, MC3T3-E1 cells were transfected with Lnc-DIF overexpression plasmid or Lnc-DIF siRNA, respectively. Lnc-DIF expression in MC3T3-E1 cells was increased by 100.5% after plasmid transfection compared with the control (Figure S2A, p < 0.001). The expression of Lnc-DIF was decreased by 53.4% after siRNA transfection (Figure S2B, p < 0.001). Transfection of both Lnc-DIF overexpression plasmid or Lnc-DIF siRNA showed no effect to cell viability (Figure S3). Upon Lnc-DIF plasmid transfection, mRNA expression levels of osteogenic marker genes *Col Iα1* and *Runx2* were dramatically downregulated by 30.1% (p < 0.001) and 40.0% (p < 0.001), respectively (Figure 2B). In addition, the ALP-positive blue-violet complexes and Alizarin Red-stained mineralized nodules were significantly decreased as well, which confirmed the effect of Lnc-DIF on inhibiting osteoblast differentiation (Figures 2A and S4A). On the other hand, the mRNA expression levels of *Col Iα1* and *Runx2* were increased by 40.2% (p < 0.001) and 40.6% (p < 0.001), respectively, after the transfection of Lnc-DIF siRNA (Figure 2D). ALP activities and mineralized nodules in the Lnc-DIF siRNA group were also significantly enhanced (Figures 2C and S4B). All these findings indicated that Lnc-DIF inhibited osteoblast differentiation.

We further determined the physiological role of Lnc-DIF in mice. Lnc-DIF overexpression plasmid was transfected into the bone marrow of mice femur with an *in vivo* transfection reagent (Entranster *In Vivo* Transfection Reagent) (Li et al., 2015, 2018). RT-PCR result confirmed that 3 days after intra-femoral injection, Lnc-DIF expression level was significantly upregulated in BMSCs (Figure S2C, p < 0.01). Bone formation marker *Alp* and *Ocn* were downregulated by 40.5% (p < 0.01) and 89.0% (p < 0.001), respectively, compared with normal control (Figure S5A). Consistently, the mineral apposition rate (MAR, an assessment of bone formation) and bone formation rate (BFR/BS) of femoral bone trabecula in Lnc-DIF transfected mice was decreased by 59.0% and 58.4%, respectively, with mineralizing surface (MS/BS) slightly decreased (Figures 2E, S5C, and S5D, p < 0.001). Moreover, microCT analysis was also made to reveal function of Lnc-DIF on trabecular bone microarchitecture; bone parameter analysis showed that bone mineral density (BMD) and bone volume to tissue volume (BV/TV) were significantly decreased in mice treated by Lnc-DIF (Figures 2G and 2I, p < 0.05).

Rescue effect of Lnc-DIF siRNA on bone formation was measured using 24-month-old mice (aging osteoporosis model). Lnc-DIF siRNA *in vivo* transfection significantly decreased Lnc-DIF expression level in BMSCs (Figure S2D, p < 0.05). The transfection also upregulated bone formation markers *Alp* and *Ocn* by 42.5% (p < 0.01) and 657.6% (p < 0.001), respectively, compared with negative control (Figure S5B). MAR and BFR/BS of si-Lnc-DIF transfection in femoral bone trabecula was increased by 49.9 and 56.7%, respectively (Figures 2F, S5C, and S5D, p < 0.001). MicroCT analysis also proved si-Lnc-DIF recovered BMD and BV/TV in aging osteoporosis mice (Figures 2H and 2J, p < 0.05). These results provided evidences that Lnc-DIF played a role in inhibiting osteoblast differentiation and bone formation *in vitro* and *in vivo*.

### Lnc-DIF was a potential competing endogenous RNA of miR-489-3p

**Figure 3:**
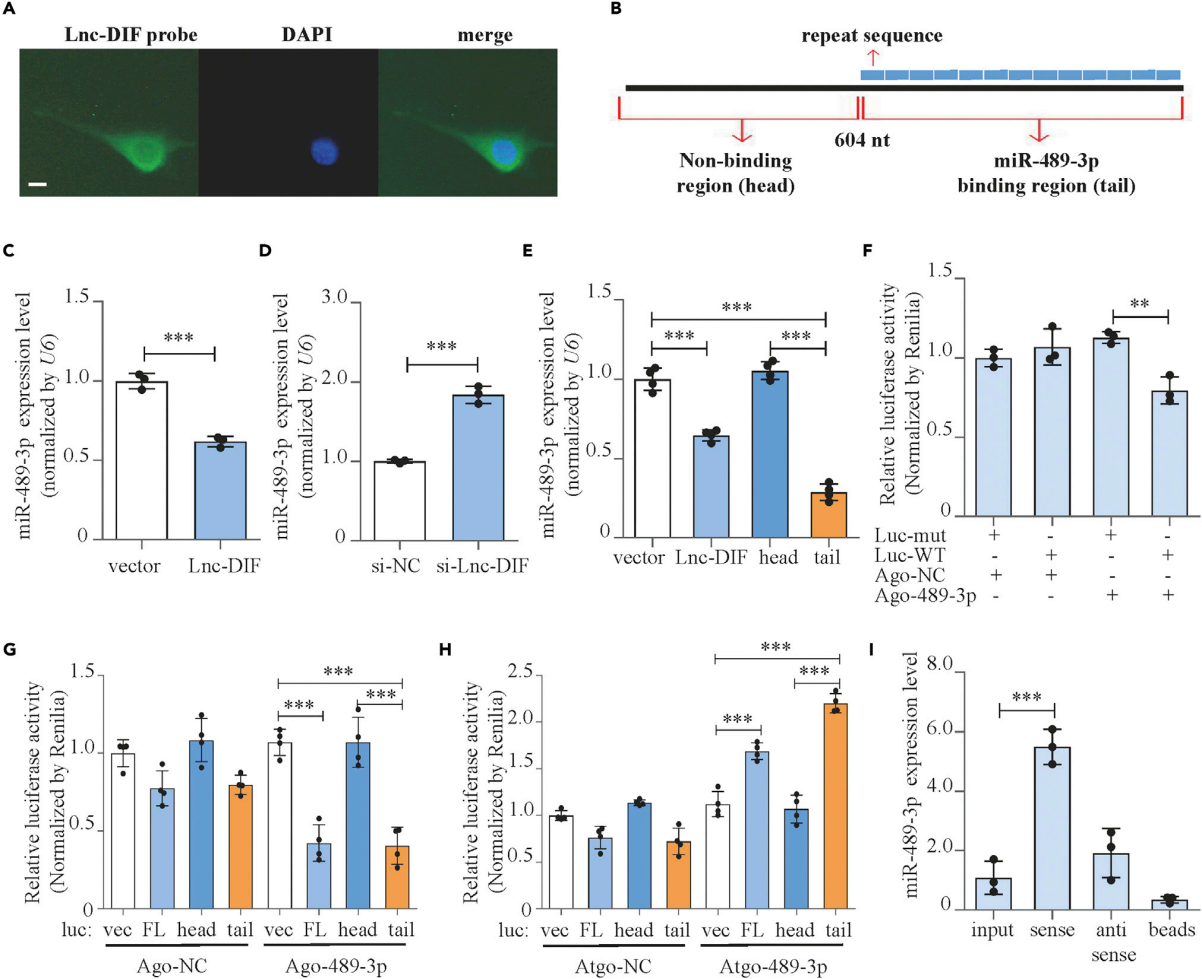
Lnc-DIF was a potential competing endogenous RNA of miR-489-3p (A) Distribution of Lnc-DIF in MC3T3-E1 cells, as detected by fluorescence *in situ* hybridization (FISH). Scale bar: 10μm. (B) Sequence structure of Lnc-DIF. (C) miR-489-3p expression levels of MC3T3-E1 cells treated with Lnc-DIF overexpression plasmid, as detected by RT-PCR (mean ± SD, ∗∗∗p < 0.001, N = 3). (D) miR-489-3p expression levels of MC3T3-E1 cells treated with Lnc-DIF siRNA, as detected by RT-PCR (mean ± SD, ∗∗∗p < 0.001, N = 3). (E) miR-489-3p expression levels of MC3T3-E1 cells treated with Lnc-DIF and Lnc-DIF region expression plasmids, as detected by RT-PCR (mean ± SD, ∗∗∗p < 0.001, N = 4). Vector: empty expression plasmid. Lnc-DIF: expression plasmid containing Lnc-DIF full length. Head: expression plasmid containing Lnc-DIF head region. Tail: expression plasmid containing Lnc-DIF tail region. (F) Binding effect of miR-489-3p and Lnc-DIF repeat sequence, as detected by luciferase reporter assay (mean ± SD, ∗∗p < 0.01, N = 3). Luc-WT: pMIR-Report luciferase reporter plasmid containing a WT miR-489-3p binding repeat sequence of Lnc-DIF. Luc-mut: luciferase reporter plasmid containing a mutant miR-489-3p binding repeat sequence of Lnc-DIF. (G and H) Binding effect of miR-489-3p and Lnc-DIF and Lnc-DIF regions, as detected by luciferase reporter assay and treated by agomiR-489-3p or antagomiR-489-3p (mean ± SD, ∗∗∗p < 0.001, N = 4). Luc-vec: empty luciferase reporter plasmid. Luc-FL: luciferase reporter plasmid containing Lnc-DIF full length. Luc-head: luciferase reporter plasmid containing Lnc-DIF head region. Luc-tail: luciferase reporter plasmid containing Lnc-DIF tail region. (I) Binding effect of miR-489-3p and Lnc-DIF, as detected by RNA pull-down and RT-PCR (mean ± SD, ∗∗∗p < 0.001, N = 3). Input: MC3T3-E1 total RNA. Sense: Lnc-DIF sense sequence. Antisense: Lnc-DIF antisense sequence. Beads: empty biotin-beads.

We further explored the mechanism on how Lnc-DIF regulated osteoblast differentiation. Previous studies have revealed that LncRNAs may act as sponges to bind miRNAs and affect their function (Salmena et al., 2011). Intracellularly, Lnc-DIF was distributed in cytoplasm, as detected by fluorescence *in situ* hybridization (FISH) (Figures 3A and S6), filling the requirement for ceRNA. The analysis of Lnc-DIF sequence revealed 13 sequence repeats in the trailing end part of Lnc-DIF. This region started at the 604th nucleotide of Lnc-DIF sequence and lasted until its end. Repeat sequence contained 53 nucleotides with the sequence “CCTGTTCTTGTTAATACTGTATACTACGCATAGATGTTATATGCAGATGTTAT,” and slight differences were observed in different repeats. Eleven out of the thirteen repeat sequences were predicted to bind with miR-489-3p by RNA22 V2 (sequences see Table 1) (Miranda et al., 2006). The 604 nucleotides that did not contain repeat sequence were designated as “head” region, whereas the repeated sequence as “tail” region (Figure 3B).

**Table 1:** Primers sequences for RT-PCR, related to STAR methods

Target gene	Sequences (5’→3′)
Lnc-DIF-Forward	CCTGTGGAGGAAGGAAGATG
Lnc-DIF-Reverse	TCAGAAGGCTGGAGAGATGG
*Col Iα1*-Forward	GAAGGCAACAGTCGATTCACC
*Col Iα1*-Reverse	GACTGTCTTGCCCCAAGTTCC
*Runx2*-Forward	CGCCCCTCCCTGAACTCT
*Runx2*-Reverse	TGCCTGCCTGGGATCTGTA
*Alp*-Forward	GTTGCCAAGCTGGGAAGAACAC
*Alp*-Reverse	CCCACCCCGCTATTCCAAAC
*Ocn*-Forward	GAAGGCAACAGTCGATTCACC
*Ocn*-Reverse	GACTGTCTTGCCCCAAGTTCC
miR-489-3p-Forward	GCGGCGGAATGACACCACATAT
miR-489-3p-Reverse	ATCCAGTGCAGGGTCCGAGG
miR-489-3p-RT	GTCGTATCCAGTGCAGGGTCCGAGGTATTCGCACTGGATACGACGCTGCC
*Smad2*-Forward	CCATCCCCGAGAACACTAACTT
*Smad2*- Reverse	TGGTGGTCGCTAGTTTCTCCAT
*Gapdh*-Forward	TGCACCACCAACTGCTTAG
*Gapdh*- Reverse	GGATGCAGGGATGATGTTC
*U6*-Forward	GTGCTCGCTTCGGCAGCACATAT
*U6*- Reverse	AAAATATGGAACGCTTCACGAA

The regulatory effect of Lnc-DIF on miR-489-3p was determined by transfecting Lnc-DIF overexpression plasmid and si-Lnc-DIF into MC3T3-E1 cells. Results showed that Lnc-DIF overexpression plasmid decreased miR-489-3p level by 38.1% (Figure 3C, p < 0.001), whereas the expression level of miR-489-3p was increased by 83.8% after transfected by si-Lnc-DIF (Figure 3D, p < 0.001).

To determine which domain of Lnc-DIF was responsible for its effect on osteoblast differentiation and miR-489-3p, expression plasmids containing Lnc-DIF head region (head) and Lnc-DIF tail region (tail) were constructed and transfected into MC3T3-E1 cells, with Lnc-DIF overexpression plasmid and empty pCDNA3.1 (+) plasmid used as positive and negative control, respectively. Transfection of Lnc-DIF head region, which had no miR-489-3p binding site, had no effect on miR-489-3p level. In contrast, Lnc-DIF tail region, which contained repeated binding sites of miR-489-3p, significantly decreased miR-489-3p (Figure 3E, p < 0.001).

Luciferase reporter assay and RNA pull-down were performed to evaluate the direct binding between Lnc-DIF and miR-489-3p. For luciferase reporter assay, reporter plasmids containing either a WT or a mutant miR-489-3p binding repeat sequence of Lnc-DIF were constructed, respectively. Reporter plasmids were transfected into MC3T3-E1 cells together with agomiR-489-3p or agomiR-NC. AgomiR-489-3p alone significantly reduced luciferase activity of the WT Lnc-DIF luciferase reporter plasmid (Figure 3F, p < 0.01). Then, luciferase reporter plasmids containing Lnc-DIF full length (Luc-FL), Lnc-DIF head region (Luc-head), and Lnc-DIF tail region (Luc-tail) were constructed and transfected into MC3T3-E1 cells, respectively, with empty pMIR-Report Luciferase plasmid (Luc-vec) used as control. AgomiR-489-3p or antagomiR-489-3p was also applied to these transfected cells with its corresponding control. Results showed that agomiR-489-3p significantly decreased luciferase activity of cells transfected with Luc-FL or Luc-tail. In the cells that were transfected with Luc-head, which had no miR-489-3p binding site theoretically, the luciferase activity was not affected (Figure 3G, p < 0.001). Conversely, antagomiR-489-3p significantly increased luciferase activity of cells transfected with Luc-FL and Luc-tail. No differences were observed for cells transfected with Luc-head (Figure 3H, p < 0.001). As for RNA pull-down, interactions of miR-489-3p with both sense and antisense Lnc-DIF were detected, with empty biotin beads (beads) and total RNA (input) as control. Result revealed that Lnc-DIF sense sequence sequestered most of miR-489-3p, compared with other groups (Figure 3I, p < 0.001).

Regulatory effects of Lnc-DIF to other miRNAs were also investigated. Osteogenic miRNAs miR-20a-5p and miR-210-3p were both predicted to bind with the repeat sequence of Lnc-DIF. Results showed that si-Lnc-DIF had no effect on the expression levels of miR-20a-5p and miR-210-3p. Lnc-DIF overexpression plasmid, as well as plasmid containing Lnc-DIF tail region only showed very slight inhibition to miR-20a-5p and miR-210-3p levels (Figure S7). All these results suggested that Lnc-DIF might act as a ceRNA of miR-489-3p; it binded with miR-489-3p through its tail region and downregulates intracellular level of miR-489-3p.

### Lnc-DIF inhibited osteoblast differentiation and bone formation through sequestering miR-489-3p

**Figure 4:**
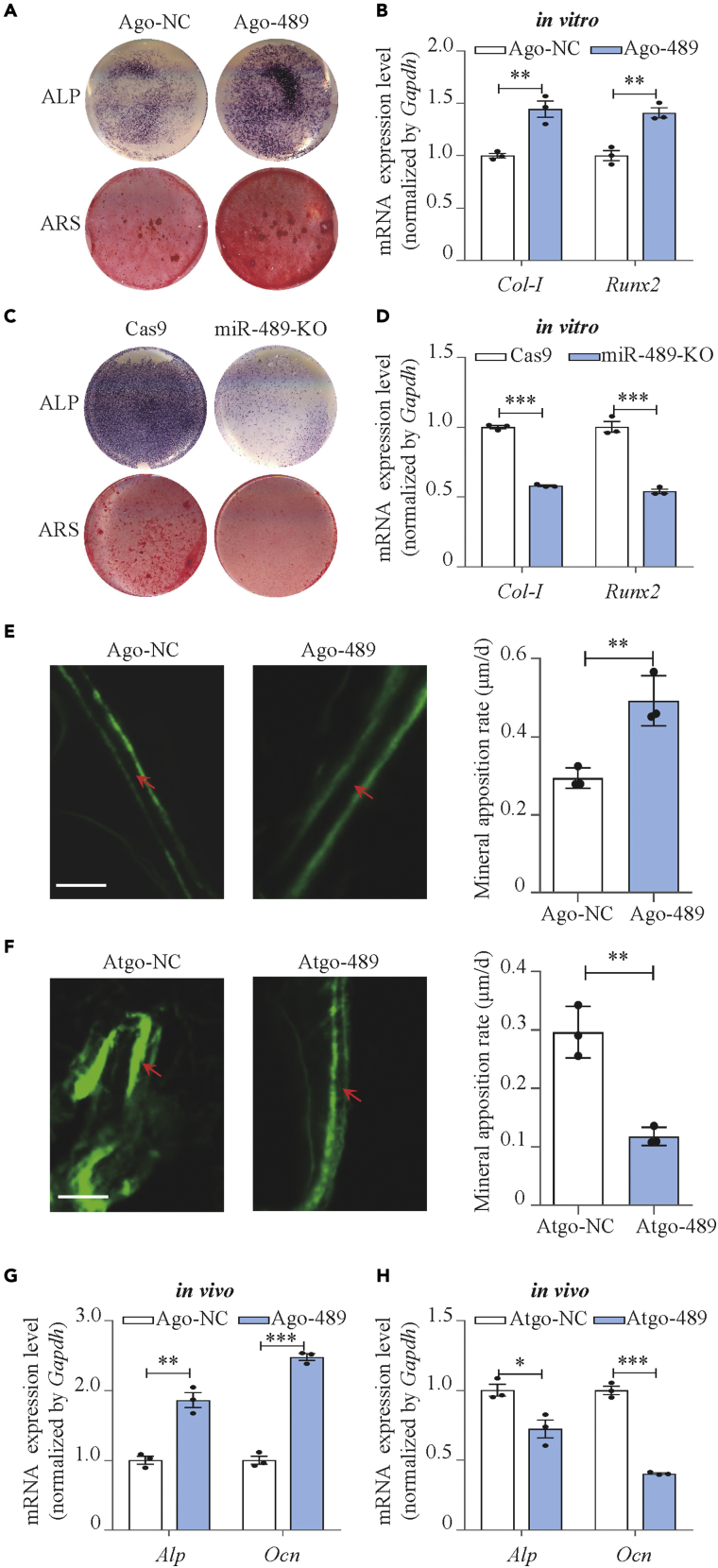
miR-489-3p promoted osteoblast differentiation and bone formation (A) Alp and Alizarin red staining of MC3T3-E1 cell treated with agomiR-489-3p (compared with negative control agomir), as detected by Alp staining and Alizarin red staining. Alp: results of Alp staining. Alz: results of Alizarin red staining. (B) *Col-I* and *Runx2* expression levels of MC3T3-E1 cell treated with agomiR-489-3p, as detected by RT-PCR (mean ± SD, ∗∗p < 0.01, N = 3). (C) Alp and Alizarin red staining of miR-489-3p knock out MC3T3-E1 cell (compared with blank CRISPR-Cas9 treated MC3T3-E1 cell), as detected by Alp staining and Alizarin red staining. Alp: results of Alp staining. Alz: results of Alizarin red staining. (D) *Col-I* and *Runx2* expression levels of miR-489-3p knock out MC3T3-E1 cell, as detected by RT-PCR (mean ± SD, ∗∗∗p < 0.001, N = 3). (E) Representative images showing femoral trabecular bone mineral apposition rate of C57BL/6 mice treated with agomiR-489-3p (mean ± SD, ∗∗p < 0.01, N = 3). Scale bar: 10μm. (F) Representative images showing femoral trabecular bone mineral apposition rate of C57BL/6 mice treated with antagomiR-489-3p (mean ± SD, ∗∗p < 0.01, N = 3). Scale bar: 10μm. (G) *Alp* and *Ocn* expression levels of C57BL/6 mice BMSCs treated with agomiR-489-3p, as detected by RT-PCR (mean ± SD, ∗∗p < 0.01, ∗∗∗p < 0.001, N = 3). (H) *Alp* and *Ocn* expression levels of C57BL/6 mice BMSCs treated with antagomiR-489-3p, as detected by RT-PCR (mean ± SD, ∗p < 0.05, ∗∗∗p < 0.001, N = 3).

Although miR-489 has been widely reported as an inhibitor for multiple cancers (Lin et al., 2017; Yuan et al., 2017), its function on osteoblast differentiation was still unclear. Palmieri et al. reported that miR-489 expression was increased in osteoblast-like cell line MG63 co-cultured by PerioGlas (a material that could enhance osteogenic differentiation) (Palmieri et al., 2008), which implied that miR-489 may have positive regulatory function for osteoblast differentiation. In this study, we transfected MC3T3-E1 cells with agomiR-489-3p to increase miR-489-3p expression level. Upon transfection, osteoblast differentiation markers *Col Iα1* and *Runx2* were upregulated by 44.4% (p < 0.01) and 40.9%, (p < 0.01) respectively (Figure 4B). ALP activities and mineralized nodules were also increased (Figures 4A and S8A, p < 0.01). Knocking out miR-489-3p in MC3T3-E1 cell using CRISPR-Cas9 significantly reduced the expression levels of *Col Iα1* and *Runx2* and decreased ALP activities and mineralized nodules, compared with control cells transfected by Cas9 blank plasmids (Figures 4C, 4D, and S8B, p < 0.001). These results proved that miR-489-3p promoted osteoblast differentiation.

The role of miR-489-3p on bone formation was also investigated by intra-femoral injection. Mice treated by agomiR-489-3p presented enhanced mineral apposition rate and bone formation rate as compared with normal control (Figures 4E and S9A). Bone formation markers *Alp* and *Ocn* expression were also enhanced (Figure 4G). Consistently, the mice treated by antagomiR-489-3p showed decreased mineral apposition rate, bone formation rate, and bone formation marker expression (Figures 4F, 4H, and S9B).

**Figure 5:**
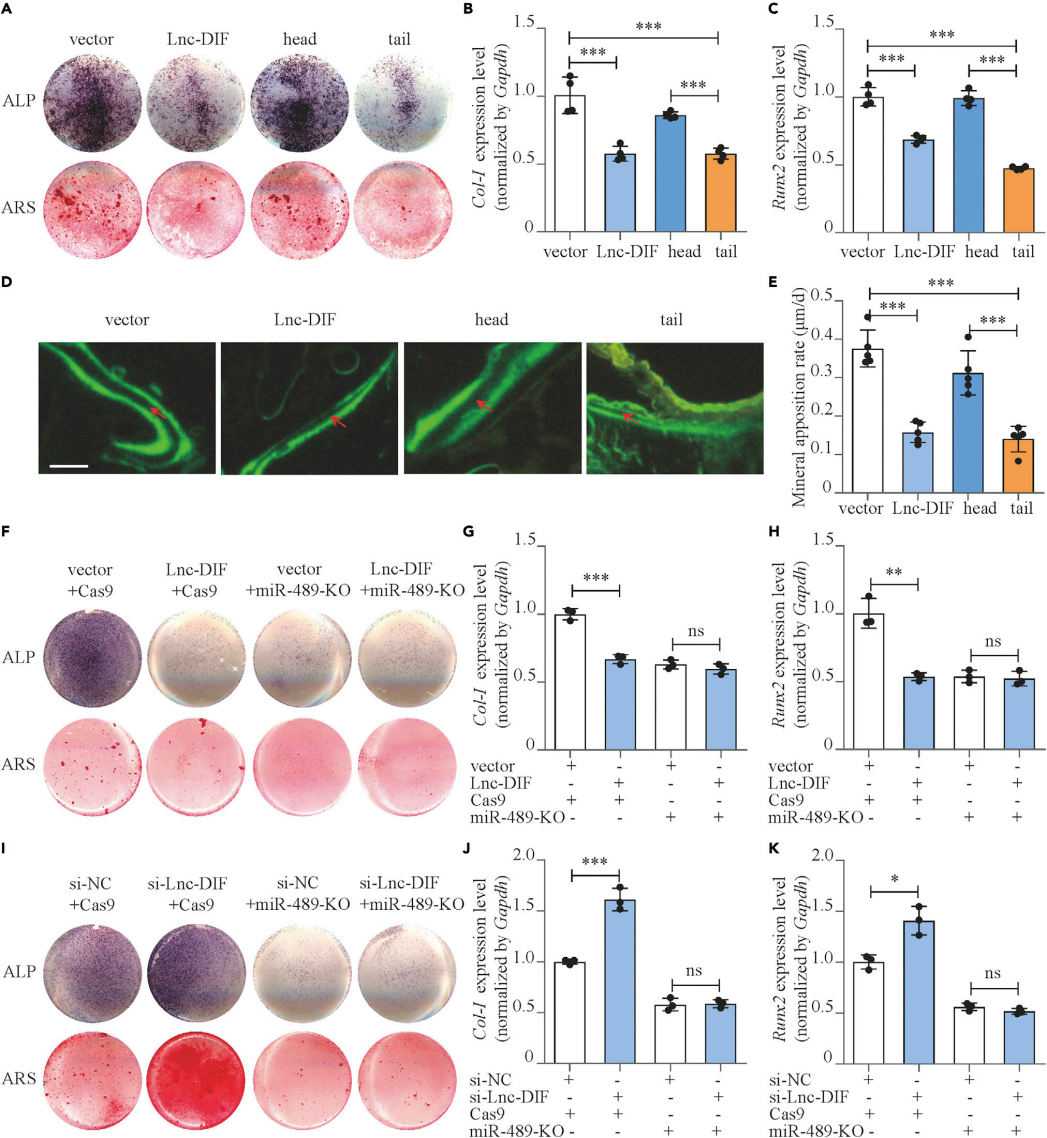
Lnc-DIF inhibited osteoblast differentiation and bone formation through sequestering miR-489-3p (A) Alp and Alizarin red staining of MC3T3-E1 cell treated with Lnc-DIF and Lnc-DIF region expression plasmids, as detected by Alp staining and Alizarin red staining. Alp: results of Alp staining. Alz: results of Alizarin red staining. Vector: empty expression plasmid. Lnc-DIF: expression plasmid containing Lnc-DIF full length. Head: expression plasmid containing Lnc-DIF head region. Tail: expression plasmid containing Lnc-DIF tail region. (B and C) *Col-I* and *Runx2* expression levels of MC3T3-E1 cells treated with Lnc-DIF and Lnc-DIF region expression plasmids, as detected by RT-PCR (mean ± SD, ∗∗∗p < 0.001, N = 4). (D) Representative images showing femoral trabecular bone mineral apposition rate of C57BL/6 mice treated with Lnc-DIF and Lnc-DIF region expression plasmids. Scale bar: 10μm. (E) Femoral trabecular bone mineral apposition rates of C57BL/6 mice treated with Lnc-DIF and Lnc-DIF region expression plasmids (mean ± SD, ∗∗∗p < 0.001, N = 5). (F) Alp and Alizarin red staining of miR-489-3p knock out MC3T3-E1 cell treated with Lnc-DIF over-expression plasmids, as detected by Alp staining and Alizarin red staining. Alp: results of Alp staining. Alz: results of Alizarin red staining. (G and H) *Col-I* and *Runx2* expression levels of miR-489-3p knock out MC3T3-E1 cells treated with Lnc-DIF over-expression plasmids, as detected by RT-PCR (mean ± SD, ∗∗p < 0.01, ∗∗∗p < 0.001, N = 3). (I) Alp and Alizarin red staining of miR-489-3p knock out MC3T3-E1 cell treated with siRNA-Lnc-DIF, as detected by Alp staining and Alizarin red staining. Alp: results of Alp staining. Alz: results of Alizarin red staining. (J and K) *Col-I* and *Runx2* expression levels of miR-489-3p knock out MC3T3-E1 cells treated with siRNA-Lnc-DIF, as detected by RT-PCR (mean ± SD, ∗p < 0.05, ∗∗∗p < 0.001, N = 3).

Subsequently, to further verify the necessity of the miR-489-3p binding site for the function of Lnc-DIF, expression plasmids with Lnc-DIF head and tail region were transfected to MC3T3-E1 cells, and their effects on osteoblast differentiation were investigated. *Col Iα1* and *Runx2* mRNA expressions in Lnc-DIF tail-region-transfected cells were decreased by 33.1% (p < 0.001) and 52.0% (p < 0.001), respectively, compared with cells transfected by Lnc-DIF head region (Figures 5B and 5C). ALP activities and mineralized nodules in Lnc-DIF tail-region-transfected cells were also significantly diminished (Figures 5A and S10). The results indicated that Lnc-DIF inhibited osteoblast differentiation by binding with miR-489-3p via its tail region.

Functions of Lnc-DIF head and tail region were further tested *in vivo*. RT-PCR result revealed that after Lnc-DIF tail region injection into the bone marrow of mice femur, miR-489-3p was decreased by 57.6% (Figure S11A, p < 0.001), and bone formation markers *Alp* and *Ocn* in BMSCs were downregulated by 41.8% (p < 0.001) and 85.8% (p < 0.001), respectively, compared with Lnc-DIF head region (Figure S11B and S11C). The MAR and BFR/BS of femoral bone trabecula in Lnc-DIF tail-region-transfected mice were decreased by 56.9% and 54.3%, respectively, compared with Lnc-DIF head region (Figures 5D, 5E, and S12, p < 0.01, p < 0.001). The results indicated that Lnc-DIF tail region inhibited bone formation through binding with miR-489-3p.

Moreover, we transfected Lnc-DIF overexpression plasmid and si-Lnc-DIF into miR-489-3p knock out MC3T3-E1 cells (miR-489-KO), with CRISPR-Cas9-treated cells as control. For control cells, when Lnc-DIF overexpression plasmid was introduced, osteoblast differentiation marker gene as well as ALP activities and mineralized nodules were decreased. However, Lnc-DIF overexpression plasmid had no effect on miR-489-KO cells (Figures 5F–5H and S13A). The transfection of si-Lnc-DIF promoted osteoblast differentiation of control cells, yet had negative effect on miR-489-KO cells (Figures 5I–K and S13B). All these results demonstrated that Lnc-DIF might act as a ceRNA of miR-489-3p, and it sequester miR-489-3p by its tail region and counteract the positive effect of miR-489-3p to osteoblast differentiation and bone formation.

### MiR-489-3p promoted osteoblast differentiation by targeting SMAD2

**Figure 6:**
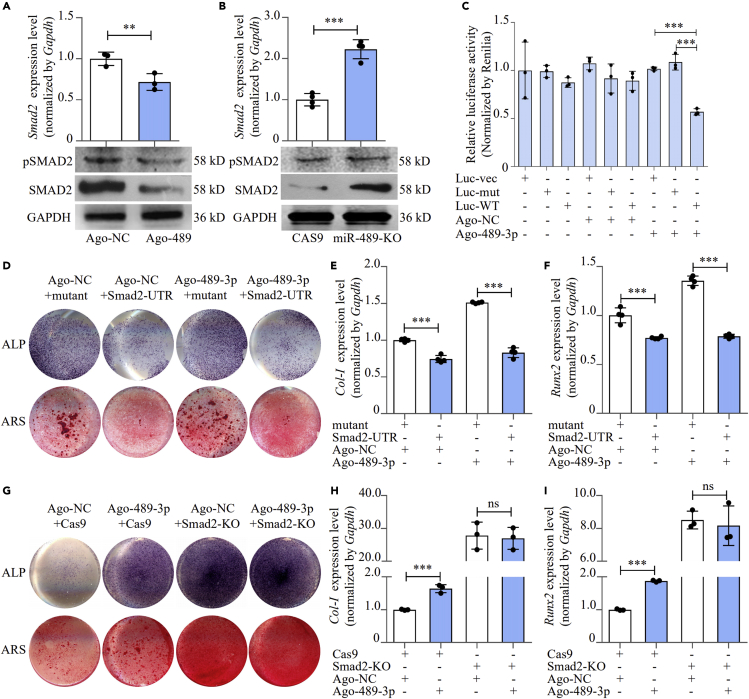
miR-489-3p promoted osteoblast differentiation by targeting SMAD2 (A) SMAD2 and phosphorylated SMAD2 levels of MC3T3-E1 cells treated with agomiR-489-3p, as detected by RT-PCR and western blot (mean ± SD, ∗∗p < 0.01, N = 3). (B) SMAD2 expression levels of miR-489-3p knock out MC3T3-E1 cells, as detected by RT-PCR and western blot (mean ± SD, ∗∗∗p < 0.001, N = 3). (C) Binding effect of miR-489-3p and *Smad2*-3′UTR, as detected by luciferase reporter assay and treated by agomiR-489-3p (mean ± SD, ∗∗∗p < 0.001, N = 3). Luc-vec: empty luciferase reporter plasmid. Luc-mut: luciferase reporter plasmid containing mutant *Smad2* 3′UTR. Luc-WT: luciferase reporter plasmid containing wild-type *Smad2* 3′UTR. (D) Alp and Alizarin red staining of MC3T3-E1 cell treated with *Smad2* 3′UTR plasmid and agomiR-489-3p, as detected by Alp staining and Alizarin red staining. Alp: results of Alp staining. Alz: results of Alizarin red staining. Mutant: expression plasmid containing mutant miR-489-3p-*Smad2* 3′UTR binding site sequence. *Smad2*-UTR: expression plasmid containing wild-type miR-489-3p-*Smad2* 3′UTR binding site sequence. (E and F) *Col-I* and *Runx2* expression levels of MC3T3-E1 cell treated with *Smad2* 3′UTR plasmid and agomiR-489-3p, as detected by RT-PCR (mean ± SD, ∗∗∗p < 0.001, N = 4). (G) Alp and Alizarin red staining of SMAD2 knock out MC3T3-E1 cell treated with agomiR-489, as detected by Alp staining and Alizarin red staining. Alp: results of Alp staining. Alz: results of Alizarin red staining. (H and I) *Col-I* and *Runx2* expression levels of SMAD2 knock out MC3T3-E1 cell treated with agomiR-489, as detected by RT-PCR (mean ± SD, ∗∗∗p < 0.001, N = 3).

It was previously reported that miR-489-3p targeted SMAD3 (Wu et al., 2016), and SMAD2/3 was an inhibitor of osteoblast differentiation (Matsumoto et al., 2012). In this study, we for the first time determined the regulatory effect of miR-489-3p on SMAD2/3 and results showed that agomiR-489-3p significantly downregulated SMAD2 mRNA and protein level and agomiR-489-3p also downregulated Ser465/Ser467 phosphorylated SMAD2 level (Figure 6A), whereas antagomiR-489-3p had opposite effect (Figure 6B). This regulatory effect on SMAD2 was also investigated by intra-femoral injection of both agomiR-489-3p and antagomiR-489-3p. AgomiR-489-3p enhanced *Smad2* expression and SMAD2 activity as compared with agomiR-NC (Figures S14A and S14C). On the contrary, antagomiR-489-3p reduced *Smad2* expression and SMAD2 activity *in vivo* (Figures S14B and S14D).

Binding of miR-489-3p to *Smad2* -3′UTR was also determined by luciferase reporter assay. The luciferase reporter plasmids that have either a wild-type *Smad2* 3′UTR (Luc-WT) or a *Smad2* 3′UTR containing mutant sequences (Luc-mut) of the miR-489-3p binding site were constructed and transfected to MC3T3-E1 cells along with AgomiR-489-3p or antagomiR-489-3p. Results exhibited that luciferase activity was significantly decreased by agomiR-489-3p in cells transfected by Luc-WT, and enhanced by antagomiR-489-3p, revealing the binding effect of miR-489-3p to *Smad2* -3′UTR (Figures 6C and S15).

To verify that SMAD2 served as mediator between miR-489-3p and osteoblast differentiation, we constructed *Smad2*-UTR plasmid that expressed miR-489-3p-*Smad2* 3′UTR binding sequence (*Smad2*-UTR), with plasmid expressing mutant binding sequence (mutant) as control. *Smad2*-UTR and mutant plasmid were transfected to MC3T3-E1 cells along with agomiR-489-3p or agomiR-NC. After transfection, osteoblast differentiation marker *Col Iα1* was downregulated by 25.7% (p < 0.001) in cells with normal miR-489-3p level (transfected by agomiR-NC), whereas *Col Iα1* was downregulated by 45.1% (p < 0.001) in high miR-489-3p cells induced by agomiR-489-3p (Figure 6E). For *Runx2*, *Smad2*-UTR decreased its expression by 23.1% (p < 0.001) in normal miR-489-3p cells and decreased its expression by 41.9% (p < 0.001) in high miR-489-3p cells (Figure 6F). ALP activities and mineralized nodules were also significantly decreased by *Smad2*-UTR (Figures 6D, S16A, and S16B). These results demonstrated that *Smad2*-UTR would inhibit the enhancing effect of miR-489-3p on osteoblast differentiation and proved that miR-489-3p inhibited osteoblast differentiation via targeting *Smad2*.

Moreover, we also knocked out *Smad2* expression in MC3T3-E1 cells by CRISPR-CAS9, and osteoblast differentiation markers *Col Iα1*, *Runx2* levels, along with ALP activities, and mineralized nodules were significantly increased (Figures S17A–S17C). SMAD2-overexpression-treated mice showed decreased mineral apposition rate, bone formation rate, and bone formation marker expression (Figures S17D and S17E). The results proved the inhibitory effect of SMAD2 on osteoblast differentiation and bone formation. The *Smad2* knockout MC3T3-E1 cells (SMAD2-KO) were then treated by agomiR-489-3p or agomiR-NC, with blank CRISPR-Cas9-treated cells as control. Results showed that in control cells, agomiR-NC significantly enhanced differentiation markers *Col Iα1*, *Runx2* expression, ALP activities, and mineralized nodules. However, in SMAD2-KO cells, no significant differences were found in osteoblast differentiation between agomiR-489-3p and agomiR-NC treatment (Figures 6G–6I, S16C, and S16D). SMAD2-KO cells were also treated by antagomiR-489-3p or antagomiR-NC. AntagomiR-489-3p inhibited osteoblast differentiation of control cells, but did not affect SMAD2-KO cells (Figure S18). All these results indicated that miR-489-3p promoted osteoblast differentiation through targeting SMAD2.

### Lnc-DIF regulated SMAD2 through miR-489-3p

**Figure 7:**
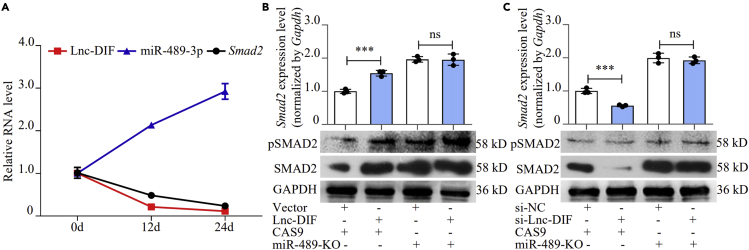
Lnc-DIF regulated SMAD2 through miR-489-3p (A) Expression levels of Lnc-DIF, miR-489-3p, and *Smad2* during the osteoblast differentiation process of MC3T3-E1 cells, as detected by RT-PCR. (B) SMAD2 and phosphorylated SMAD2 levels of miR-489-3p knock out MC3T3-E1 cells treated with Lnc-DIF overexpression plasmids, as detected by RT-PCR and western blot (mean ± SD, ∗∗∗p < 0.001, N = 3). (C) SMAD2 and phosphorylated SMAD2 levels of miR-489-3p knock out MC3T3-E1 cells treated with siRNA-Lnc-DIF, as detected by RT-PCR and western blot (mean ± SD, ∗∗∗p < 0.001, N = 3).

The relationship between Lnc-DIF, miR-489-3p, and SMAD2 was further investigated. We measured mRNA expression levels of Lnc-DIF, miR-489-3p, and *Smad2* in MC3T3-E1 at the 0, 12^th^, and 24^th^ day of osteoblast differentiation induction. Lnc-DIF and *Smad2* expression levels decreased with the progress of osteoblast differentiation, whereas expression levels of miR-489-3p were increased during this progress (Figure 7A). The results indicated a positive relationship between Lnc-DIF and *Smad2* expression but negative correlation between Lnc-DIF and miR-489-3p expression.

Regulatory effects of Lnc-DIF to SMAD2 were also investigated by transfecting Lnc-DIF overexpression plasmid and si-Lnc-DIF into miR-489-3p knockout MC3T3-E1 cells (miR-489-KO), along with CRISPR-Cas9 control cells. In control cells, transfection of Lnc-DIF overexpression plasmid caused a 54.6% increment of *Smad2* mRNA expression (p < 0.001) as well as protein level and phosphorylated SMAD2 level. This result demonstrated that Lnc-DIF overexpression enhanced SMAD2 expression and activity. However, in miR-489-KO MC3T3-E1 cells, SMAD2 and phosphorylated SMAD2 levels were not dramatically changed by Lnc-DIF (Figure 7B). On the contrary, transfection of si-Lnc-DIF resulted in decrease of *Smad2* mRNA expression by 44.1% (p < 0.001) as well as the protein, suggesting again the positive regulatory effect of Lnc-DIF on SMAD2. However, silencing Lnc-DIF did not influence SMAD2 expression and activity in miR-489-KO cells (Figure 7C). All these results suggested that Lnc-DIF upregulated SMAD2, and the positive regulation effect was dependent on miR-489-3p.

Subsequently, we transfected expression plasmids with Lnc-DIF head and tail region to MC3T3-E1 cells. Expression level of SMAD2 and phosphorylated SMAD2 levels in Lnc-DIF tail-region-transfected cells were both increased compared with cells transfected by Lnc-DIF head region, and the *in vivo* result also supported our conclusion (Figure S19).

### Rescue effect of Lnc-DIF siRNA on bone formation in OVX mice

**Figure 8:**
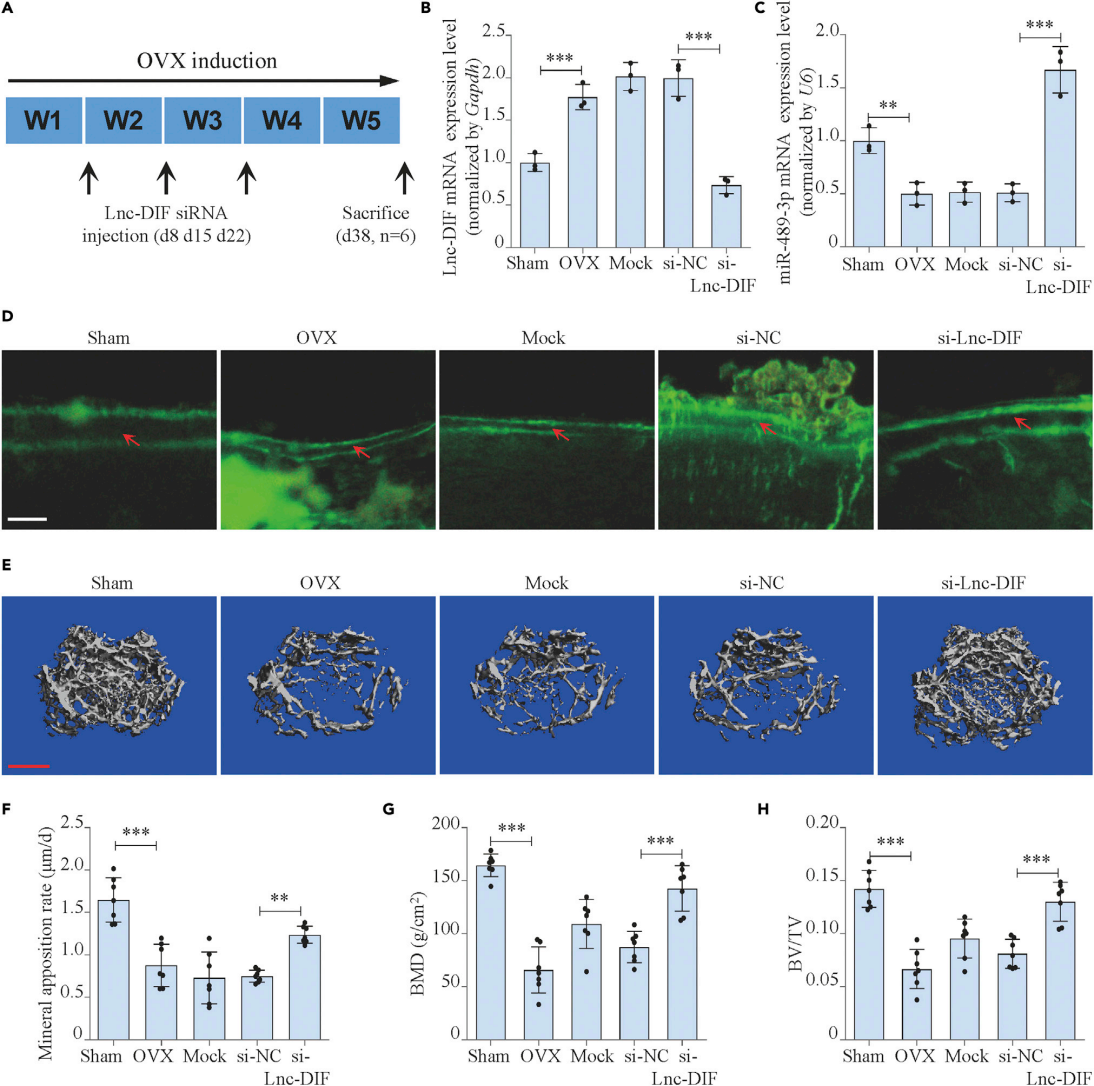
Rescue effect of Lnc-DIF siRNA on bone formation in OVX mice (A) Schematic graph showing experimental design. (B and C) Lnc-DIF and miR-489-3p expression levels of C57BL/6 mice BMSCs after OVX treatment and siRNA-Lnc-DIF treatment, as detected by RT-PCR (mean ± SD, ∗∗p < 0.01, ∗∗∗p < 0.001, N = 3). Sham: sham OVX operation group. OVX: OVX group. Mock: osteoblast-targeting delivery system control group. si-NC: negative control si-RNA treated group. si-Lnc-DIF: siRNA-Lnc-DIF treated group. (D) Representative images showing femoral cortical bone mineral apposition rate of C57BL/6 mice after OVX treatment and siRNA-Lnc-DIF treatment. Scale bar: 10μm. (E) Representative images showing femoral trabecular microarchitecture of C57BL/6 mice after OVX treatment and siRNA-Lnc-DIF treatment, as detected by micro-CT. Scale bar: 500μm. (F–H) Femoral cortical bone mineral apposition rates, bone mineral density (BMD), and bone volume to tissue volume (BV/TV) of C57BL/6 mice after OVX treatment and siRNA-Lnc-DIF treatment (mean ± SD, ∗∗p < 0.01, ∗∗∗p < 0.001, N = 7).

In view of the results presented earlier, we moved forward to investigate the rescue effect of Lnc-DIF siRNA on osteoporosis mice. The OVX mice were separately treated with si-Lnc-DIF or si-NC carried by an osteoblast-targeting delivery system, which was a targeting system involving dioleoyl trimethylammonium propane (DOTAP)-based cationic liposomes attached to six repetitive sequences of aspartate, serine, and serine [(AspSerSer)(6)] for delivering siRNAs specifically to bone-formation surfaces (Wang et al., 2013). The animals received three consecutive injections via tail vein every week during the OVX process (Figure 8A). We found that the expression of Lnc-DIF in mice BMSCs was enhanced by OVX surgery but was strongly downregulated upon siRNA treatment (Figure 8B). In the contary, miR-489-3p expression was downregulated in BMSCs of OVX mice and recovered by si-Lnc-DIF injection (Figure 8C). In addition, the MAR of femoral cortical bone was decreased by 48.3% (p < 0.001) after the ovariectomy, whereas it was recovered by 31.0% (p < 0.01) after the exposing to si-Lnc-DIF; BFR/BS showed a similar tendency as MAR (Figures 8D, 8F, and S20). Similarly, microCT results showed that si-Lnc-DIF revealed the deteriorated changes of trabecular bone mass and trabecular microarchitecture caused by OVX (Figure 8E). Further, bone parameter analysis showed that bone mineral density (BMD), bone volume to tissue volume (BV/TV), and trabecular number (Tb.N) were significantly decreased in OVX mice compared with sham, whereas this decrease was revealed by si-Lnc-DIF. Conversely, the trabecular separation (Tb.Sp) was higher in OVX groups than sham group and lower after treating with si-Lnc-DIF (Figures 8G, 8H, and S20). These data suggested that si-Lnc-DIF could rescue the minus consequence of menopause, therefore promoting mice bone formation.

## Discussion

Osteoporosis is a high incidence bone disease in aging population. The main symptoms of osteoporosis include reduced bone mass, deterioration of bone microstructure, and decreased bone strength, which may increase the risk of fractures (Clarke and Khosla, 2010). Osteoporosis has become an emerging threat to human health in recent years. The current therapeutic strategies for osteoporosis are still limited. Therefore, developing a therapeutic target for osteoporosis would have great significance for prevention and treatment of osteoporosis.

LncRNAs play an important role in regulating bone formation. An increasing number of research proved that LncRNAs can regulate osteoblast differentiation and bone formation (Feng et al., 2020; He et al., 2019; Li et al., 2017, 2018, 2019, 2020; Mulati et al., 2020; Peng et al., 2018a, 2018b; Wang et al., 2018, 2019, 2020; Wu et al., 2018; Wu et al., 2019; Yi et al., 2019; Yin et al., 2019; Yuan et al., 2019; Zhang et al., 2019). For example, Li et al. reported that LncRNA H19 promoted bone formation by activating Erk and Wnt/β-catenin signaling pathway (Li et al., 2017, 2018). Mulati et al. found that LncRNA Crnde deficiency resulted in significant osteoporosis by establishing the Crnde knock out mice, and Crnde enhanced Wnt/β-catenin signaling pathway (Mulati et al., 2020). He et al. discovered LncRNA ODIR1, interacted with F-box protein 25, and inhibited the osteogenic differentiation of human umbilical-cord-derived mesenchymal stem cells (Li et al., 2017).

An essential mechanism is that LncRNAs can function as sponges for miRNAs and further inhibit miRNA function (Salmena et al., 2011; Thomson and Dinger, 2016). For example, Wang et al. recently showed that LncRNA ODSM binds with miR-139-3p and promoted osteoblast function and bone formation through miR-139-3p/ELK1 axis (Wang et al., 2020). Feng et al. evaluated the LncRNA XIST's function in periodontal ligament stem cells and showed that XIST sponged miR-214-3p to promote osteogenic differentiation (Feng et al., 2020). Zhang et al. reported LncRNA NEAT1/miR-29b-3p/BMP1 Axis promoted osteogenic differentiation of human bone-marrow-derived mesenchymal stem cells (Zhang et al., 2019). However, functional LncRNAs as ceRNA might have low efficient. In this study, we have identified long noncoding RNA Lnc-DIF that functioned as an inhibitor of osteoblast differentiation and bone formation. Lnc-DIF contained a special structure of repeat sequences in its trailing end, and this repeat sequence can sequester multiple miR-489-3p and efficiently inhibit the positive effect of miR-489-3p/SMAD2 axin on osteoblast differentiation and bone formation. The study provided an important hint that some LncRNAs may contain endogenous repeats and potentially sequester miRNAs. Manipulating the expression levels of these LncRNAs might be a potential therapy for aging and postmenopausal osteoporosis.

On the basis of our previous study, we have further screened LncRNA AK138929 negatively in association with osteogenic differentiation and therefore named it as Lnc-DIF (differentiation inhibiting factor). The functions of Lnc-DIF were further investigated. Experiments *in vitro* confirmed that Lnc-DIF inhibited osteoblast differentiation (Figure 2), same as the function of Lnc-DIF *in vivo*. So far, only a few studies reported the function of LncRNA in bone formation. In our previous studies, siRNA of AK016739 and AK045490 were injected subcutaneously over the calvarial surface, and the calvarial bone formation was analyzed (Li et al., 2019; Yin et al., 2019). However, aging and postmenopausal osteoporosis mostly occur in weight-bearing bones, especially femur and tibia. In Yuan’s study, siRNA was administrated into the bone marrow of mice femur to investigate the function of PGC1β-OT1 on femoral bone formation (Yuan et al., 2019). In this study, Lnc-DIF overexpression plasmid or Lnc-DIF siRNA was injected into medullary cavity of femur. The MAR of femoral bone trabecula proved that Lnc-DIF inhibited bone formation (Figure 2).

LncRNAs could bind with miRNAs and further sequester them. This kind of LncRNAs was defined as ceRNAs (Salmena et al., 2011; Thomson and Dinger, 2016). So far, most LncRNAs that function as ceRNA only have one binding site for one sort of miRNA, which means one LncRNA can only sequester one miRNA. This resulted in low efficiency and low specificity. In our study, we found 13 sequence repeats with slight differences in the trailing end part of Lnc-DIF, 11 of 13 repeats were predicted to bind with miR-489-3p, which suggested that Lnc-DIF may act as an efficient miRNA sponge, and one single molecular of Lnc-DIF would potentially sequester 11 miR-489-3p. The plasmids containing Lnc-DIF repeat sequence were constructed, and the repeat sequence was proved to bind with miR-489-3p and would efficiently decrease miR-489-3p level, which was similar to the function of Lnc-DIF full length (Figure 3). Regulatory effects of Lnc-DIF to other miRNAs were also investigated. miR-20a-5p and miR-210-3p were both predicted binding with Lnc-DIF repeat sequence and promoting osteogenic differentiation. Result showed both Lnc-DIF and its repeat sequence do not affect miR-20a-5p and miR-210-3p levels. The result proved the specificity of Lnc-DIF repeat sequence binding and regulating miR-489-3p.

The function of miR-489 had been widely reported as an inhibitor for cancer (Lin et al., 2017; Yuan et al., 2017), whereas only limited researchers reported its osteogenic functions (Palmieri et al., 2008). In this study, we have firstly proved that miR-489-3p enhanced osteoblast differentiation (Figure 4). With the absence of miR-489-3p, neither Lnc-DIF overexpression nor Lnc-DIF knockdown could affect osteoblast differentiation (Figure 5). These results confirmed that the function of Lnc-DIF as an osteoblast differentiation inhibitor relied on its sequestering of miR-489-3p. We also firstly proved SMAD2 as the target gene of miR-489-3p. SMAD2 inhibited osteoblast differentiation (Matsumoto et al., 2012), and Lnc-DIF enhanced SMAD2 level through miR-489-3p (Figure 7). These results revealed the mechanism of Lnc-DIF that function as a ceRNA that sequestered miR-489-3p and impeded its inhibiting effect to SMAD2 and therefore inhibited osteoblast differentiation and bone formation.

RNA-based therapy has become an emerging tendency in exploring more methods to rescue bone metabolic diseases. The development of RNA delivery system or bone-specific aptamer would transport RNA sequences to its target region with high efficiency (Liang et al., 2015; Liu et al., 2015; Wang et al., 2013; Zhang et al., 2012). The technique had made the siRNA of Lnc-DIF as a potential therapy of osteoporosis. We investigated the rescue effect of si-Lnc-DIF to postmenopausal osteoporosis by intravenously injecting si-Lnc-DIF together with an osteoblast-targeting delivery system. The delivery system was developed previously and have been used to deliver nucleic acid specially to osteoblast with low side effects and toxicity (Wang et al., 2013; Zhang et al., 2012). The results showed that si-Lnc-DIF significantly enhanced bone formation and trabecular microarchitecture in osteoporosis mice (Figure 8), which further proved Lnc-DIF as a potential therapeutic target of osteoporosis.

As an important regulatory factor to bone formation, LncRNAs has received increasing attention of related researchers. However, due to its complicated mechanisms and low homology between different species, LncRNAs were seldom considered as a direct therapy for osteoporosis. In this study, a special repeat sequence was found in Lnc-DIF. This repeat sequence was proved to bind with miR-489-3p and inhibit osteoblast differentiation and bone formation. This implied us that there are more LncRNAs that exist as endogenous sponges for osteogenic miRNAs. Manipulating the expression levels of these sponges would have an amplified effect for regulating their target miRNA levels and would efficiently regulate bone formation. A representative example was our study, in which we utilized the osteoblast-targeting delivery system (Wang et al., 2013) to transport si-Lnc-DIF and treated ovariectomized osteoporosis mice. Moreover, one more miR-489-3p sponge has been detected in human genome, and its function has already been determined *in vitro*. This discovery provide an ideality to RNA-based therapy and might be developed as potential therapeutic strategy of osteoporosis in our further studies.

Taken together, this study has identified Lnc-DIF as an inhibitor for osteoblast differentiation and bone formation. Lnc-DIF was also identified as a ceRNA that sequestered multiple miR-489-3p by its repeat sequence and inhibited osteoblast differentiation and bone formation through manipulating miR-489-3p/SMAD2 axis. This study has revealed a special LncRNA function as high efficient miRNA sponge, which provided more ideas and potential therapeutic strategies for osteoporosis.

### Limitations of the study

Our study discovered a long noncoding RNA that inhibited bone formation via miR-489-3P/SMAD2; it provided potential therapeutic insight for RNA-based therapy for osteoporosis. However, Lnc-DIF is a mice-derived LncRNA inhibiting bone formation, which means neither Lnc-DIF sequence nor its siRNA would be used as a therapeutic RNA for human osteoporosis. However, this study has provided us the idea that screening LncRNAs with special repeat sequences in human genome would be a potential way for osteoporosis diagnosis and treatment.

## STAR★Methods

### Key resources table

**Table:** 

REAGENT or RESOURCE	SOURCE	IDENTIFIER
**Antibodies**		

SMAD2 Rabbit polyclonal antibody	Proteintech	Cat# 12570-1-AP, RRID:AB_2193037
Ser465/Ser467 phosphorylated SMAD2 Rabbit polyclonal antibody	Bioss	Cat# bs-3419R, RRID:AB_10880886
GAPDH Rabbit polyclonal antibody	Proteintech	Cat# 10494-1-AP, RRID:AB_2263076

**Bacterial and virus strains**		

pCDNA3.1+	miaolingbio, Wuhan, China	P0157
pMIR-Report Luciferase plasmid	miaolingbio, Wuhan, China	P0471
pRL-TK Renilla plasmid	Dr. Pengsheng Zheng (Xi’an Jiaotong University, Xi’an, China)	N/A
PNL1.1 nanoluc Report Luciferase plasmid	Promega	N1001
lentiCRISPR-V2	Dr. Jie Zhao (Tianjin medical University, Tianjin, China)	Addgene 52961

**Chemicals, peptides, and recombinant proteins**		

Alizarin red S	Sigma	A5533
Calcein green	Sigma	C0875

**Critical commercial assays**		

Entranster^TM^*In Vivo* Transfection Reagent	Engreen Biosystem Co. Ltd., Beijing, China	18668-11-2
Engreen Entranster^TM^ H4000 Reagent	Engreen Biosystem Co. Ltd., Beijing, China	4000-6
dual-luciferase reporter assay system	Promega	E1910
BCIP/NBT Alkaline Phosphatase Color Development Kit	Beyotime Biotechnology, Shanghai, China	C3206
Fluorescent *In Situ* Hybridization Kit	RiboBio, guangzhou, China	C10910

**Deposited data**		

Supplemental Table S1 LncRNA microarray data	This paper; Mendeley Data	E-MTAB-11426
Supplemental Table S2 mRNA microarray data	This paper; Mendeley Data	E-MTAB-11425

**Experimental models: Cell lines**		

Murine preosteoblast MC3T3-E1 cell line	Dr. Hong Zhou (The University of Sydney, Sydney, Australia)	N/A
Isolatied mice bone marrow mesenchymal stem cells	This paper	N/A

**Oligonucleotides**		

Lnc-DIF siRNA 5’-GGCAUGUAAUCUCUAGACAdTdT-3’ (sense). 3’-dTdTCCCGUACAUUAGAGAUCUGU-5’ (antisense).	This paper	N/A
miR-489-3p agomir 5’-AAUGACACCACAUAUAUGGCAGC-3’ (sense). 3’-UGCCAUAUAUGUGGUGUCAUUUU-5’ (antisense).	This paper	N/A
miR-489-3p antagomir 5’-GCUGCCAUAUAUGUGGUGUCAUU-3’	This paper	N/A

**Recombinant DNA**		

Lnc-DIF full length sequence	https://www.ncbi.nlm.nih.gov/nuccore/AK138929	N/A

**Software and algorithms**		

Image-Pro Plus 6.0	Image-Pro Plus	N/A
Image J	National Institutes of Health	N/A
GraphPad Prism 6.0	GraphPad	N/A

### Resource availability

#### Lead contact

Further information and requests for resources should be directed to and will be fulfilled by the lead contact, Chong Yin (yinchong42@nsmc.edu.cn).

#### Materials availability

All reagents used in this study will be made available on request to the lead contact.

### Experimental model and subject details

Murine preosteoblast MC3T3-E1 cell line was generously provided by Dr. Hong Zhou (The University of Sydney, Sydney, Australia). MC3T3-E1 cell line were cultured in osteoblast culture medium, which contains Alpha Modified Eagle’s Medium (α-MEM, Gibco, 11900-024, Carlsbad, CA) supplemented with 10% fetal bovine serum (FBS; Biological Industries, 04-001-1A, Kibbutz Beit Haemek, Israel), 1% L-glutamine (Sigma, G8540, St Louis, MO), 1% penicillin (Amresco, 0242, Solon, OH) and streptomycin (Amresco, 0382, Solon, OH). Cell cultures were maintained at a humidified, 37°C, 5% CO_2_ incubator (Thermo Fisher Scientific, Waltham, MA). For osteogenic differentiation treatment, MC3T3-E1 cells at density of 100% were induced by osteogenic medium with α-MEM, 10% FBS, 1% β-glycerophosphate (Sigma, G9422), 1% ascorbic acid (Sigma, A7631) and 1% L-glutamine. The cell cultures were maintained at 37°C with 5% CO_2_, and medium was replaced every 2 days.

Aging and ovariectomized (OVX) mice was adopted to construct the osteoporosis model. All mice were purchased from the Laboratory Animal Center of the Fourth Military Medical University (Xi’an, China). For aging mice model, 6-month-old male C57BL/6 mice were maintained under standard animal housing conditions (12-h light, 12-h dark cycles and free access to food and water). Mice which were kept until 18 month old were selected as aging group. Mice were euthanized and femurs were collected and processed for bone marrow mesenchymal stem cells isolation.

For OVX mouse model, 2-month-old female C57BL/6 mice were maintained under standard animal housing conditions. The mice were ovariectomized or sham-operated at 3 months of age. Mice were euthanized 38 days after surgery (4 months of age) and femurs were collected. Euthanasia was performed by CO_2_. Mice were euthanized and femurs were collected and processed for bone marrow mesenchymal stem cells isolation. All animal experiments were performed in accordance with the recommendation of “the Guiding Principles for the Care and Use of Laboratory Animals” (the Institutional Experimental Animal Committee of Northwestern Polytechnical University, Xi’an, China) and all experimental procedures were approved by the Institutional Experimental Animal Committee of Northwestern Polytechnical University, Xi’an, China. For all procedures involving animals, all efforts were made to reduce the number of the mice used and their suffering.

For Lnc-DIF plasmid *in vivo* transfection, female C57BL/6 mice (4 month old) were randomly divided into four groups (vector, Lnc-DIF, head, tail). For each group, mice were injected into medullary cavity of femur with plasmids formulated with Entranster^TM^ *In Vivo* Transfection Reagent (Engreen Biosystem Co. Ltd., 18668-11-2, Beijing, China) at the dosage of 40 μL according to the manufacturer’s instructions. All mice received the same standard diet during the experimental period. 3 mice from each groups were euthanized 3 days after treatment and femurs from mice were processed for BMSC isolation and RT-PCR. All other Mice were euthanized 15 days after treatment and femurs were processed for histomorphometric analyses (Li et al., 2015, 2018).

For Lnc-DIF siRNA *in vivo* transfection, male aging C57BL/6 mice (24 month old) were randomly divided into two groups (si-NC, si-Lnc-DIF). In the si-Lnc-DIF group, mice were injected into medullary cavity of femur with plasmids formulated with Entranster^TM^ *In Vivo* Transfection Reagent (Engreen Biosystem Co. Ltd., 18668-11-2, Beijing, China) at the dosage of 40 μL according to the manufacturer’s instructions. In the si-NC group, mice were injected with negative control siRNA mixed with the same technique. All mice received the same standard diet during the experimental period. 3 mice from each groups were euthanized 3 days after treatment and femurs from mice euthanized 15 days after OVX were processed for BMSC isolation and RT-PCR. All other Mice were euthanized 12 days after treatment and femurs were processed for histomorphometric analyses and MicroCT.

For miR-489-3p agomir and antagomir *in vivo* transfection, 4 month old female C57BL/6 mice were injected into medullary cavity of femur by the same method as Lnc-DIF injection. 3 mice from each groups were euthanized 3 days after treatment and femurs from mice were processed for BMSC isolation and RT-PCR or SMAD2 luciferase reporter assay. Other 3 Mice were euthanized 15 days after treatment and femurs were processed for histomorphometric analyses.

### Method details

#### Isolation of bone marrow mesenchymal stem cells (BMSCs)

After sacrifice, mice femoral bones were immediately harvested and attached soft tissues were carefully removed. Bone marrow was washed and collected by flushing several times with phosphate buffered saline (PBS) using a 25G syringe needle. The collected PBS with bone marrow were centrifuged (1200 g, 8 min) and mechanically dissociated by culture medium (α-MEM, Gibco supplemented with 10% fetal bovine serum, 1% L-glutamine, 1% penicillin and streptomycin) using a 29G syringe needle. Then, the suspension was cultured in a 60 mm plate for 3 hours (37°C, 5% CO_2_) followed by carefully wash with culture medium. Cells were cultured for another 36 hours with culture medium changed every 12 hours. The cells were transferred into a new plate as the 1st-passage cells. Third passage cells were used for characterization and experiments.

#### mRNA, LncRNA microarray

To investigate the mRNAs and LncRNAs changed with MACF1 (a cytoskeletal protein positively regulate osteoblast differentiation and bone formation via multiple osteogenic transcription factors), RNA from MACF1 knockdown MC3T3-E1 preosteoblasts and negative control cells were used for mRNA and LncRNA microarray. Microarray was performed at RiboBio Co. Ltd. (Yin et al., 2019). The fold change of each deferentially expressed mRNA and lncRNA was obtained by log_2_ (normalized intensity of treat/normalized intensity of control). Quantile normalization method was used and average of repeated data from the same sample was taken. The p-values were calculated with ANOVA Method.

#### Screening of osteogenic LncRNAs

The LncRNA-array results may contain hundreds or thousands of lncRNAs, and these lncRNAs were screened by certain standards. LncRNAs that longer than 3500 nt might be hard to insert into vectors and transfect into cells. While, LncRNAs that shorter than 800 nt might be hard to design siRNAs, which made it unable to determine its function. Thus, we only select LncRNAs with length between 800-3500nt. Then, to select lncRNAs that highly correlated with osteogenic differentiation, only LncRNAs that were significantly changed in MACF1-knockdown MC3T3-E1 cell were chosen. RT-PCR was established in normal MC3T3-E1 cells first, and the chosen LncRNAs with a CT value higher than 42 were weeded out. Remaining LncRNAs were selected for co-expression network analysis.

#### Co-expression network analysis

Co-expression network analysis was adopted to screen the LncRNAs correlated with osteogenic mRNAs (Yin et al., 2019). Overexpressed LncRNAs in the microarray data were briefly screened by over-expression fold change and LncRNA length. Then the co-expression metrices were created by computing Pearson’s correlation coefficient (PCC) between each screened LncRNA and mRNAs related to osteogenic pathways (Wnt signaling pathway, bmp signaling pathway, TGF-beta signaling pathway, HIF-1 signaling pathway) . The average correlation values of each LncRNA were calculated and LncRNAs with most significant correlation value was selected (Shannon et al., 2003).

#### Real time PCR

RT-PCR was used to assess expression levels of selected LncRNAs and osteogenic genes. Total RNA was extracted from mouse tissues or cultural cells using Trizol reagent. Mice tissues were harvested and grinded with liquid nitrogen and then digested by Trizol reagent. 1μg of total RNA was used for cDNA synthesis using one step PrimeScript RT reagent kit (TaKaRa, RR037A, Dalian, China). Quantitative PCR amplification was performed using the Thermal Cycler C-1000 Touch system (BIO-RAD CFX Manager, Hercules, CA) and SYBR Premix Ex TaqII kit (TaKaRa, RR820A). For LncRNA and mRNA, *Gapdh* was used as internal control gene, as for miRNAs, *U6* was used as internal control gene. The quantitative PCR reaction conditions included initial denaturation step at 95°C for 30s, followed by 40 cycles at 95°C for 10s, 60°C for 30s, and 72°C for 5s. Data were calculated using the comparative Ct method (2^-ΔΔCt^) and expressed as fold change compared with corresponding control. Primers (sequences see Table 1) were synthesized by Sangon Int (Shanghai, China).

#### Western blot

For detection of protein levels, Western blot analysis was performed as previously described (Yin et al., 2018). Cultural cells were washed three times by cold PBS and then digested by cell lysis buffer (Beyotime, P0013, Haimen, China) with 1% protease inhibitor cocktail (Calbiochem, 539134, Darmstadt, Germany) on ice for 30mins. Protein concentrations were analysed by BCA protein assay kit (Thermo Fisher Scientific, 23225). An equal amount of protein for each sample was subjected to SDS-PAGE using 5% stacking gel and 12% separating gel, 120V, 1h and transferred (400mA, 2h) to nitrocellulose filter membranes (Pall, 66485, Port Washington, NY). Membranes were blocked with 5% skimmed milk (BD Biosciences, 232100, Franklin Lakes, NJ) for 1 hour at room temperature, and then incubated with primary antibodies at 4°C overnight with the following primary antibodies: SMAD2 (Rabbit pAb, 1:1000; Proteintech, 12570-1-AP, Hubei, China), Ser465/Ser467 phosphorylated SMAD2 (Rabbit pAb, 1:700; Bioss, bs-3419R, Beijing, China) and GAPDH (Rabbit pAb, 1:1000; Proteintech, 10494-1-AP). Blots were then incubated with HRP-labeled secondary antibody (1:2000; CWBIO, CW0103, Beijing, China) and visualized using chemiluminescence detection system (Thermo Fisher Scientific, NCI5080). Protein bands were exposed to X-ray film (Kodak, 6535876, Rochester, NY). GAPDH was adopted as internal control.

#### Transfection of the Lnc-DIF plasmid and siRNA

Lnc-DIF full length sequence (https://www.ncbi.nlm.nih.gov/nuccore/AK138929), its head region, and its tail region were synthesized by TsingKe Int (Beijing, China) and inserted into pCDNA3.1 (+) plasmid (miaolingbio, P0157, Wuhan, China), respectively. For transfection *in vitro*, MC3T3-E1 cells were seeded in a 6-well plate at 1.5×10^5^ cells per well and were transfected with Lnc-DIF plasmid (or its head/tail region plasmid) by Engreen Entranster^TM^ H4000 Reagent (Engreen, 4000-6, Beijing, China) according to the manufacturer’s instructions, using empty pCDNA3.1 (+) plasmid as normal control. The over-expression of Lnc-DIF in MC3T3-E1 cells were confirmed by RT-PCR 48 hours after the transfection.

Lnc-DIF siRNA transfection was performed to knockdown Lnc-DIF. For *in vitro* transfection, MC3T3-E1 cells were seeded in a 6-well plate at 1×10^5^ cells per well and were transfected with 100nM Lnc-DIF-siRNA (RiboBio, Guangzhou, China) by lipofectamine 2000 (Invitrogen, 11668-030). 4h after transfection, the serum-free medium was replaced by the antibiotic-free growth medium (α-MEM with 10% FBS, and 1% L-glutamine). After 36 hours culture, MC3T3-E1 cells were harvested for RT-PCR analysis of Lnc-DIF level. For both Lnc-DIF plasmid and siRNA transfection, cell viability was detected by MTT 48 hours after the transfection.

Lnc-DIF-siRNA sequences were:

5’-GGCAUGUAAUCUCUAGACAdTdT-3’ (sense strand).

3’-dTdTCCCGUACAUUAGAGAUCUGU-5’ (antisense strand).

#### Transfection of the miR-489-3p agomir and antagomir

MiR-489-3p agomir and antagomir transfection was performed to enhance or inhibit miR-489-3p expression. MC3T3-E1 cells were seeded in a 6-well plate at 1×10^5^ cells per well and were transfected with 200nM miR-489-3p agomir or antagomir (RiboBio, Guangzhou, China) in α-MEM, respectively. 6h after transfection, the medium was replaced by the antibiotic-free growth medium (α-MEM with 10% FBS, and 1% L-glutamine). After 42h culture, MC3T3-E1 cells were harvested for RT-PCR analysis of miR-489-3p level (Wang et al., 2013).

miR-489-3p agomir and antagomir sequences were:

5’-AAUGACACCACAUAUAUGGCAGC-3’ (agomir sense strand).

3’-UGCCAUAUAUGUGGUGUCAUUUU-5’ (agomir antisense strand).

5’-GCUGCCAUAUAUGUGGUGUCAUU-3’ (antagomir strand).

#### Alkaline phosphatase staining and alizarin red staining

ALP staining and Alizarin red staining were performed to determine osteoblast differentiation. Alkaline phosphatase (ALP) of osteoblasts was stained by BCIP/NBT Alkaline Phosphatase Color Development Kit (Beyotime Biotechnology, C3206, Shanghai, China) according to the manufacturer’s instruction. Briefly, cells were washed with PBS (pH7.4) and fixed in 10% buffered formaldehyde. The formaldehyde were washed and then cells were stained using 5-bromo-4-chloro-3-indolyl phosphate (BCIP)/nitro blue tetrazolium (NBT) solution. The staining reaction was stopped by washing with distilled water and the cell staining was imaged by CanoScan 9000F Mark II scanner (Canon, Tokyo, Japan) and Relative Staining Area of ALP was analyzed by Image-Pro Plus 6.0 software.

For Alizarin red staining, cells were cultured in osteogenic medium. The staining was carried out on day 25, cells were washed with PBS and then stained in 0.5% Alizarin red S (PH 4.0, Sigma, A5533) for 30mins. After washes with tap water, the plates were dried and scanned with CanoScan 9000F Mark II scanner and Relative Staining Area of Alizarin red S was analyzed by Image-Pro Plus 6.0 software.

#### Bone histomorphometric analyses

Bone histomorphometric analysis was performed to investigate the effect of Lnc-DIF on bone formation. To measure mineral appositional rate, double calcein labeling was performed by intraperitoneal injection with calcein green (30mg/kg body weight, Sigma, C0875) in the time sequence of 11 and 3 days before euthanasia for specimen collection. Collected femur samples were fixed with 4% paraformaldehyde, dehydrated by 50% sucrose, and embedded in OCT (Leica, 14020108926, Wetzlar, Germany). Transverse cyrosections (6μm in thickness) were made by a freezing-microtome (Leica, CM1100) and slides were examined with a fluorescent microscope (NEXCOPE NIB900, Ningbo, China). Bone dynamic histomorphometric analyses for MAR, BFR/BS, MS/BS were performed using image analysis software (Image J, National Institutes of Health, Bethesda, MD) (Ushiku et al., 2010).

#### MicroCT analysis

To investigate the effect of Lnc-DIF on bone formation, the femur was scanned by the microCT system (version 6.5, viva CT40, SCANCO Medical, Switzerland) and the distal femoral metaphysis was analyzed. Briefly, the femur was fixed overnight in 70% ethanol and analyzed by microCT. Images of femurs were reconstructed and calibrated at the isotropic voxel size of 10.5 μm, respectively (70 kVp, 114 μA, 200 ms integration time, 260 thresholds, 1200 mg HA/cm3). Using the Scanco evaluation software, regions of interest (ROIs) were defined for trabecular parameters. The entire femora were reoriented with the mid-diaphysis parallel to the z-axis, and bone length was measured as the distance between the most proximal and distal transverse plans containing the femur. Starting from the most proximal aspect of the growth plate, the trabeculae region on 100 consecutive slices were selected. The trabeculae were analyzed by manually contouring excluding the cortical bone for three-dimensional reconstruction (sigma = 1.2, supports = 2 and threshold = 200) to calculate the following trabeculae parameters including bone mineral density (BMD), bone volume to tissue volume (BV/TV), trabecular number (Tb.N), trabecular separation (Tb.Sp) for trabecular microarchitecture.

#### Fluorescence *in situ* hybridization

Fluorescence *in situ* hybridization (FISH) was used to determine Lnc-DIF distribution in osteoblasts by using Fluorescent *In Situ* Hybridization Kit (RiboBio, C10910, Guangzhou, China) according to the manufacturer’s instruction. MC3T3-E1 cells were seeded in a 24-well plate at 1×10^4^ cells per well. Cells were washed with PBS (pH7.4), fixed in 10% buffered formaldehyde, and permeated with 0.5% Triton X-100 tris buffer saline (TBS) at room temperature. The cells were blocked with pri-hybridization buffer (with 1% blocking solution) for 30 minutes at 37°C. Then, the cells were incubated with hybridization buffer containing 250nM Lnc-DIF probe (5’- AACATCTGCGTATAACATCTAT -3’) overnight at 37°C. The cells were further washed with SSC buffer and DAPI buffer was used to counterstain cell nuclei for 10 minutes. Finally, the cells were washed by PBS and examined with a fluorescent microscope (Nikon 80i, Tokyo, Japan). EGFP (green fluorescence) and DAPI (blue fluorescence) were excited at a wavelength of 488 nm and 405 nm, respectively.

#### RNA-pulldown

RNA-RNA-pulldown was used to investigate the integration of Lnc-DIF and miR-489-3p. Sense and antisense of Lnc-DIF were synthesized and binded to biotin by Pierce™ RNA 3' End Desthiobiotinylation Kit (Thermo, 20163), respectively. The RNA binded with biotin were further incubated with Pierce™ Protein A/G Magnetic Beads (Thermo, 88802) for 1 hour at 4°C, respectively, with beads binded with biotin as normal control. After applying to magnet set and removing the supernatants, beads were washed with Low Salt Immune Complex wash buffer (0.1% SDS, 1% Triton X-100, 2 mM EDTA, 20 mM Tris-HCl, pH 8.1, 150 mM NaCl), high salt immune complex wash buffer (0.1% SDS, 1% Triton X-100, 2 mM EDTA, 20 mM Tris-HCl, pH 8.1, 500 mM NaCl), LiCl immune complex wash buffer (0.25 M LiCl, 1% IGEPAL-CA630, 1% deoxycholic acid, 1 mM EDTA, 10 mM Tris, pH 8.1), and TE Buffer (10 mM Tris-HCl, 1 mM EDTA, pH 8.0), successively. 1×10^8^ MC3T3-E1 cells were cultured, lysed, and total RNA was extracted and applied to the beads. Beads were then washed and removed from the magnet set. Binded RNA were washed and miR-489-3p from Lnc-DIF-biotin-beads (sense), Lnc-DIF antisense-biotin-beads (antisense), empty biotin-beads (beads), and total RNA (input) were detected.

#### Luciferase reporter assay

To detect interaction between Lnc-DIF and miR-489-3p, mutant binding sequence of Lnc-DIF and miR-489-3p (Luc-mut), unmutanted binding sequence of Lnc-DIF and miR-489-3p (Luc-wt), Lnc-DIF full length sequence (Luc-FL), its head region (Luc-head), and its tail region (Luc-tail) were synthesized by TsingKe Int (Beijing, China) and inserted into pMIR-Report Luciferase plasmid (miaolingbio, P0471 ,Wuhan, China), respectively, with empty pMIR-Report Luciferase plasmid (Luc-vec) as control. Internal control pRL-TK Renilla plasmid were generously provided by Dr. Pengsheng Zheng (Xi’an Jiaotong University, Xi’an, China). MC3T3-E1 cells were seeded in a 6-well plate at 1×10^5^ cells per well and transfected with 200nM miR-489-3p agomir or antagomir in α-MEM, respectively. 6 hours after transfection, the medium was replaced by antibiotic-free growth medium (α-MEM with 10% FBS, and 1% L-glutamine). After 3h culture, reporter plasmids were co-transfected with pRL-TK Renilla plasmid by Engreen Entranster^TM^ H4000 Reagent according to the manufacturer’s instructions. Cells were cultured for 72 h and luciferase assays were performed with the dual-luciferase reporter assay system (Promega, E1910, Fitchburg, WI) according to the manufacturer’s instruction. Luminescent signals were quantified by microplate reader (Synergy, USA), and each value from the firefly luciferase constructs was normalized by Renilla luciferase assay (Yin et al., 2019).

To detect interaction between miR-489-3p and *Smad2*, mutant binding sequence of *Smad2* 3’UTR and miR-489-3p (Luc-mut) and unmutanted binding sequence of *Smad2* 3’UTR and miR-489-3p (Luc-wt) were synthesized by TsingKe Int (Beijing, China) and inserted into pMIR-Report Luciferase plasmid, respectively, MC3T3-E1 cell transfection and luciferase assays were performed as mentioned above.

To detect activities of SMAD2, motif sequence of SMAD2 was synthesized by TsingKe Int (Beijing, China) and inserted into PNL1.1 nanoluc Report Luciferase plasmid. The plasmid along with agomir/antagomir or Lnc-DIF plasmids were injected into medullary cavity of 4 month old female C57BL/6 mice femur. 3 mice from each groups were euthanized 3 days after treatment and femurs from mice were processed for BMSC isolation. Luciferase assays were performed as mentioned above.

#### CRISPR-Cas9

CRISPR-Cas9 was used to construct SMAD2 and miR-489-3p knock out osteoblast cell line. CRISPR-Cas9 plasmid lentiCRISPR-V2 was generously provided by Dr. Jie Zhao (Tianjin medical University, Tianjin, China). sgRNA sequences of SMAD2 and miR-489-3p have been inserted to the sgRNA scafford region of plasmid, respectively. Plasmids were electroplated into MC3T3-E1 cells (1×10^7^ per well) by Neon Transfection System (Invitrogen, Carlsbad, CA) according to manufacturer’s instructions, respectively. Blank plasmid lentiRISPR-V2 without sgRNA inserted to the sgRNA scafford region was used as normal control. After the electroporation (1800 V, 30 ms), cells were seeded into a 6-well plate with 7mL α-MEM. Adherent cells were washed by 2mL α-MEM two times and medium was changed with antibiotic-free culture medium 6 hours after the electroporation. After culture for 48h, medium was changed to the selective growth medium supplemented with 3.5 μg/mL puromycin (Solarbio, P8230, Beijing, China) and cells were cultured for 7 days (Yin et al., 2018). Screened cells were used as SMAD2 and miR-489-3p knock out osteoblast cells.

#### Therapeutic si-Lnc-DIF in OVX mice

To investigate Lnc-DIF siRNA therapeutic effect, OVX mice were randomly divided into four groups (OVX, Mock, si-NC, si-Lnc-DIF). The transfection was performed 8, 15, and 22 days after OVX , respectively. In the si-Lnc-DIF group, mice received tail vein injection of si-Lnc-DIF (10 mg/kg body weight) with the osteoblast-targeting delivery system (Wang et al., 2013; Zhang et al., 2012). In the si-NC group, mice were injected with negative control siRNA mixed with the osteoblast-targeted delivery system. In the Mock group, mice received the same volume of normal saline mixed with osteoblast-targeted delivery system alone. In the OVX group, mice were given no treatment. All mice received the same standard diet during the experimental period. All mice of the baseline group and 3 mice from other groups were euthanized 17 days after OVX treatment. All other mice were euthanized 38 days after OVX and femurs were collected. The femurs from mice euthanized 17 days after OVX were processed for BMSC isolation and RT-PCR, femurs from mice euthanized 38 days after OVX were processed for microCT and histomorphometric analyses (n = 6/group) (Wang et al., 2013; Yin et al., 2019; Zhang et al., 2012).

### Quantification and statistical analysis

All experiments were independently repeated at least three times with each done in triplicate. The statistical analyses of the data were performed with GraphPad Prism version 6.0 software (GraphPad Software Inc, La Jolla, CA), and ordinary one-way ANOVA was used for variance analysis with 3 or more groups. Significance between two groups were determined using Student’s t-test. The data are presented as mean ± standard deviation (SD). *P* values < 0.05 were considered statistically significant for all comparisons.

## Data Availability

Microarray data is deposited in a publicly accessible data base and the accession code for this data is E-MTAB-11426 (https://www.ebi.ac.uk/arrayexpress/experiments/E-MTAB-11426) for LncRNA microarray data, and mRNA microarray data is E-MTAB-11425 (https://www.ebi.ac.uk/arrayexpress/experiments/E-MTAB-11425). Data reported in this paper will be shared by the lead contact upon request. This paper does not report original code. Any additional information required to reanalyze the data reported in this paper is available from the lead contact upon request

## Supplemental information

Document S1. Figures S1–S21

Table S1. mRNA microarray data, related to STAR Methods

Table S2. LncRNA microarray data, related to STAR Methods

## References

[bib1] Batista P., Chang H. (2013). Long noncoding RNAs: cellular address codes in development and disease. Cell.

[bib2] Clarke B., Khosla S. (2010). Physiology of bone loss. Radiol. Clin. North Am..

[bib3] Feng Y., Wan P., Yin L.L. (2020). Long noncoding RNA X-inactive specific transcript (XIST) promotes osteogenic differentiation of periodontal ligament stem cells by sponging MicroRNA-214-3p. Med. Sci. Monit..

[bib4] Geisler S., Coller J. (2013). RNA in unexpected places: long non-coding RNA functions in diverse cellular contexts. Nat. Rev. Mol. Cell Biol.

[bib5] Hassan M., Tye C., Stein G., Lian J. (2015). Non-coding RNAs: epigenetic regulators of bone development and homeostasis. Bone.

[bib6] He S., Yang S., Zhang Y., Li X., Gao D., Zhong Y., Cao L., Ma H., Liu Y., Li G. (2019). LncRNA ODIR1 inhibits osteogenic differentiation of hUC-MSCs through the FBXO25/H2BK120ub/H3K4me3/OSX Axis. Cell Death Dis.

[bib7] Hung T., Wang Y., Lin M., Koegel A., Kotake Y., Grant G., Horlings H., Shah N., Umbricht C., Wang P. (2011). Extensive and coordinated transcription of noncoding RNAs within cell-cycle promoters. Nat. Genet..

[bib8] Li B., Liu J., Zhao J., Ma J., Jia H., Zhang Y., Xing G., Ma X. (2017). LncRNA-H19 Modulates Wnt/β-catenin signaling by targeting Dkk4 in hindlimb unloaded rat. Orthop. Surg..

[bib9] Li B., Zhao J., Ma J., Li G., Zhang Y., Xing G., Liu J., Ma X. (2018). Overexpression of DNMT1 leads to hypermethylation of H19 promoter and inhibition of Erk signaling pathway in disuse osteoporosis. Bone.

[bib10] Li C., Cheng P., Liang M., Chen Y., Lu Q., Wang J., Xia Z., Zhou H., Cao X., Xie H. (2015). MicroRNA-188 regulates age-related switch between osteoblast and adipocyte differentiation. J. Clin. Invest.

[bib11] Li D., Tian Y., Yin C., Huai Y., Zhao Y., Su P., Wang X., Pei J., Zhang K., Yang C. (2019). Silencing of LncRNA AK045490 promotes osteoblast differentiation and bone formation via β-catenin/TCF1/Runx2 signaling axis. Int. J. Mol. Sci..

[bib12] Li G., Yun X., Ye K.S., Zhao H., An J., Zhang X., Han X., Li Y., Wang S. (2020). Long non-coding RNA-H19 stimulates osteogenic differentiation of bone marrow mesenchymal stem cells via the microRNA-149/SDF-1 axis. J. Cell Mol Med.

[bib13] Liang C., Guo B., Wu H., Shao N., Li D., Liu J., Dang L., Wang C., Li H., Li S. (2015). Aptamer-functionalized lipid nanoparticles targeting osteoblasts as a novel RNA interference-based bone anabolic strategy. Nat. Med..

[bib14] Lin Y., Liu J., Huang Y., Liu D., Zhang G., Kan H., Luca S., Zollino I., Carinci F. (2017). microRNA-489 Plays an anti-metastatic role in human hepatocellular carcinoma by targeting matrix metalloproteinase-7. Transl Oncol..

[bib15] Liu J., Dang L., Li D., Liang C., He X., Wu H., Qian A., Yang Z., Au D., Chiang M. (2015). A delivery system specifically approaching bone resorption surfaces to facilitate therapeutic modulation of microRNAs in osteoclasts. Biomaterials.

[bib16] Marie P. (1999). Cellular and molecular alterations of osteoblasts in human disorders of bone formation. Histol. Histopathol.

[bib17] Meng L., Ward A., Chun S., Bennett C., Beaudet A., Rigo F. (2015). Towards a therapy for Angelman syndrome by targeting a long non-coding RNA. Nature.

[bib18] Matsumoto Y., Otsuka F., Hino J., Miyoshi T., Takano M., Miyazato M., Makino H., Kangawa K. (2012). Bone morphogenetic protein-3b (BMP-3b) inhibits osteoblast differentiation via Smad2/3 pathway by counteracting Smad1/5/8 signaling. Mol. Cell Endocrinol.

[bib19] Miranda K., Huynh T., Tay Y., Ang Y., Tam W., Thomson A., Lim B., Rigoutsos I. (2006). A pattern-based method for the identification of MicroRNA bonding sites and their corresponding heteroduplexes. Cell.

[bib20] Mulati M., Kobayashi Y., Takahashi A., Numata H., Saito M., Hiraoka Y., Ochi H., Sato S., Ezura Y., Yuasa M. (2020). The long noncoding RNA Crnde regulates osteoblast proliferation through the Wnt/β-catenin signaling pathway in mice. Bone.

[bib21] Palmieri A., Pezzetti F., Spinelli G., Arlotti M., Avantaggiato A., Scarano A., Scapoli L., Zollino I., Carinci F. (2008). PerioGlas regulates osteoblast RNA interfering. J. Prosthodont..

[bib22] Peng S., Cao L., He S., Zhong Y., Ma H., Zhang Y., Ma H., Zhang Y., Shuai C. (2018). An overview of long non-coding RNAs involved in bone regeneration from mesenchymal stem cells. Stem Cells Int.

[bib23] Peng W., Deng W., Zhang J., Pei G., Rong Q., Zhu S. (2018). Long noncoding RNA ANCR suppresses bone formation of periodontal ligament stem cells via sponging miRNA-758. Biochem. Biophys. Res. Commun..

[bib24] Salmena L., Poliseno L., Tay Y., Kats L., Pandolfi P. (2011). A ceRNA hypothesis: the Rosetta Stone of a hidden RNA language?. Cell.

[bib25] Thomson D., Dinger M. (2016). Endogenous microRNA sponges: evidence and controversy. Nat. Rev. Genet..

[bib26] Shannon P., Markiel A., Ozier O., Baliga N., Wang J., Ramage D., Amin N., Schwikowski B., Ideker T. (2003). Cytoscape: a software environment for integrated models of biomolecular interaction networks. Genome Res..

[bib27] Tripathi V., Ellis J., Shen Z., Song D., Pan Q., Watt A. (2010). The nuclear-retained noncoding RNA MALAT1 regulates alternative splicing by modulating SR splicing factor phosphorylation. Mol. Cell.

[bib28] Ushiku C., Adams D., Jiang X., Wang L., Rowe D. (2010). Long bone fracture repair in mice harboring GFP reporters for cells within the osteoblastic lineage. J. Orthop. Res..

[bib29] Wang C., Liao Z., Xiao H., Liu H., Hu Y., Liao Q., Zhong D. (2019). LncRNA KCNQ1OT1 promoted BMP2 expression to regulate osteogenic differentiation by sponging miRNA-214. Exp. Mol. Pathol..

[bib30] Wang X., Guo B., Li Q., Peng J., Yang Z., Wang A., Li D., Hou Z., Lv K., Kan G. (2013). miR-214 targets ATF4 to inhibit bone formation. Nat. Med..

[bib31] Wang Y., Wang K., Hu Z., Zhou H., Zhang L., Wang H., Li G., Zhang S., Cao X., Shi F. (2018). MicroRNA-139-3p regulates osteoblast differentiation and apoptosis by targeting ELK1 and interacting with long noncoding RNA ODSM. Cell Death Dis.

[bib32] Wang Y., Wang K., Zhang L., Tan Y., Hu Z., Dang L., Zhou H., Li G., Wang H., Zhang S. (2020). Targeted overexpression of the long noncoding RNA ODSM can regulate osteoblast function in vitro and in vivo. Cell Death Dis.

[bib33] Wu J., Zhao J., Sun L., Pan Y., Wang H., Zhang W. (2018). Long non-coding RNA H19 mediates mechanical tension-induced osteogenesis of bone marrow mesenchymal stem cells via FAK by sponging miR-138. Bone.

[bib34] Wu Q., Han L., Yan W., Ji X., Han R., Yang J., Yuan J., Ni C. (2016). miR-489 inhibits silica-induced pulmonary fibrosis by targeting MyD88 and Smad3 and is negatively regulated by lncRNA CHRF. Sci. Rep..

[bib35] Wu Y., Jiang Y., Liu Q., Liu C. (2019). LncRNA H19 promotes matrix mineralization through up-regulating IGF1 by sponging miR-185-5p in osteoblasts. BMC Mol. Cell Biol.

[bib36] Yan K., Arfat Y., Li D., Zhao F., Chen Z., Yin C., Sun Y., Hu L., Yang T., Qian A. (2016). Structure prediction: new insights into decrypting long noncoding RNAs. Int. J. Mol. Sci..

[bib37] Yi J., Liu D., Xiao J. (2019). LncRNA MALAT1 sponges miR-30 to promote osteoblast differentiation of adipose-derived mesenchymal stem cells by promotion of Runx2 expression. Cell Tissue Res.

[bib38] Yin C., Zhang Y., Hu L., Tian Y., Chen Z., Li D., Zhao F., Su P., Ma X., Zhang G., Miao Z. (2018). Mechanical unloading reduces microtubule actin crosslinking factor 1 expression to inhibit β-catenin signaling and osteoblast proliferation. J. Cell Physiol.

[bib39] Yin C., Tian Y., Yu Y., Wang H., Wu Z., Huang Z., Zhang Y., Li D., Yang C., Wang X. (2019). A novel long noncoding RNA AK016739 inhibits osteoblast differentiation and bone formation. J. Cell Physiol.

[bib40] Yuan H., Xu X., Feng X., Zhu E., Zhou J., Wang G., Tian L., Wang B. (2019). A novel long noncoding RNA PGC1β-OT1 regulates adipocyte and osteoblast differentiation through antagonizing miR-148a-3p. Cell Death Differ..

[bib41] Yuan P., He X., Rong Y., Cao J., Li Y., Hu Y., Liu Y., Li D., Lou W., Liu M. (2017). KRAS/NF-κB/YY1/miR-489 signaling axis controls pancreatic cancer metastasis. Cancer Res..

[bib42] Zhang G., Guo B., Wu H., Tang T., Zhang B., Zheng L., He Y., Yang Z., Pan X., Chow H. (2012). A delivery system targeting bone formation surfaces to facilitate RNAi-based anabolic therapy. Nat. Med..

[bib43] Zhang Y., Chen B., Li D., Zhou X., Chen Z. (2019). LncRNA NEAT1/miR-29b-3p/BMP1 Axis promotes osteogenic differentiation in human bone marrow-derived mesenchymal stem cells. Pathol. Res. Pract..

[bib44] Zhao J., Sun B., Erwin J., Song J., Lee J. (2008). Polycomb proteins targeted by a short repeat RNA to the mouse X chromosome. Science.

