# Supplementary material for: Long noncoding RNA Lnc-DIF inhibits bone formation by sequestering miR-489-3p
Source: iScience. 2022 Feb 21;25(3):103949. doi: 10.1016/j.isci.2022.103949 (PMC8898894; doi:10.1016/j.isci.2022.103949)
Supplement: Document S1. Figures S1–S21 [file mmc1.pdf]

iScience, Volume *25*

Supplemental information

Long noncoding RNA Lnc-DIF inhibits bone formation by sequestering miR-489-3p

Chong Yin, Ye Tian, Dijie Li, Yang Yu, Shanfeng Jiang, Yimei Hou, Meng Deng, Kaiyuan Zheng, Yan Zhang, Xiaoni Deng, Zhihao Chen, Zhiping Miao, Qiang Hao, Yu Li, and Airong Qian

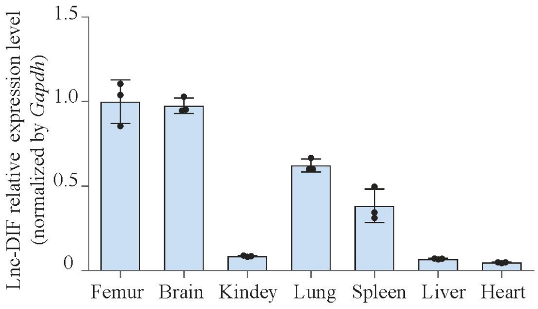

Figure S1. Expression levels of Lnc-DIF in multiple tissues of C57BL/6 mice, as detected by RT-PCR (mean ± SD, N=3). Related to Figure 1.

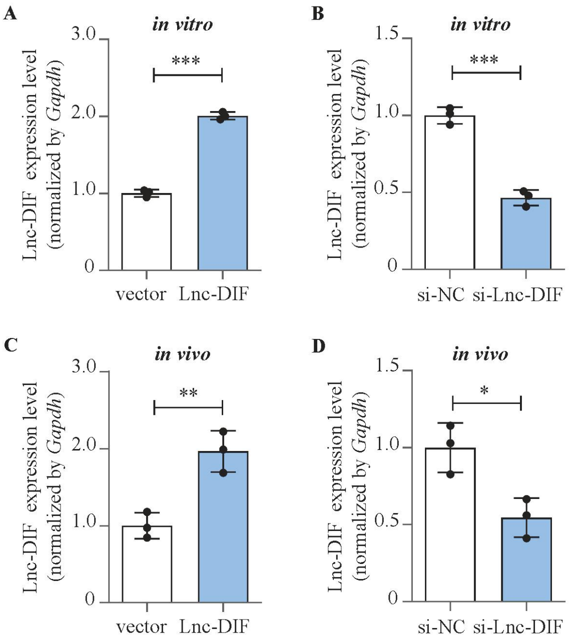

Figure S2. Effects of Lnc-DIF over-expression plasmid and siRNA-Lnc-DIF. Related to Figure 2.

A. Lnc-DIF expression levels of MC3T3-E1 cells treated with Lnc-DIF over-expression plasmid (compared with normal control), as detected by RT-PCR (mean ± SD, ***P<0.001, N=3).

B. Lnc-DIF expression levels of MC3T3-E1 cells treated with Lnc-DIF siRNA (compared with negative control siRNA), as detected by RT-PCR (mean ± SD, ***P<0.001, N=3).

C. Lnc-DIF expression levels of C57BL/6 mice treated with Lnc-DIF over-expression plasmid (compared with normal control), as detected by RT-PCR (mean ± SD, **P<0.01, N=3).

D. Lnc-DIF expression levels of 24 month aging C57BL/6 mice treated with Lnc-DIF siRNA (compared with negative control siRNA), as detected by RT-PCR (mean ± SD, *P<0.05, N=3).

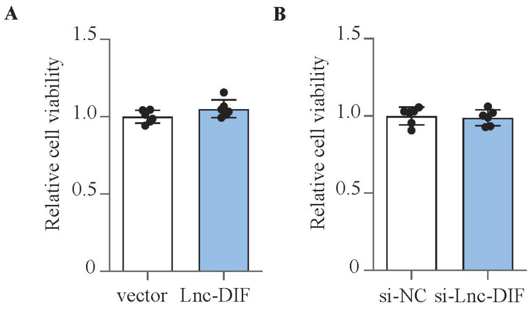

Figure S3. Effect of Lnc-DIF over-expression plasmid and siRNA-Lnc-DIF on cell viability. Related to Figure 2.

A. Viability of MC3T3-E1 cells treated with Lnc-DIF over-expression plasmid (compared with normal control), as detected by MTT (mean ± SD, N=3).

B. Viability of MC3T3-E1 cells treated with Lnc-DIF siRNA (compared with negative control siRNA), as detected by MTT (mean ± SD, N=3).

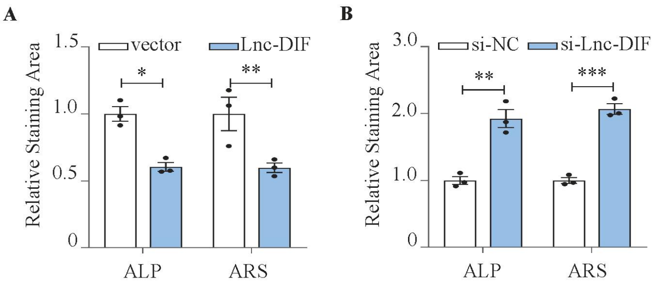

Figure S4. Relative staining areas of osteoblast treated by Lnc-DIF. Related to Figure 2.

A. Relative staining areas of ALP and Alizarin red staining of MC3T3-E1 cell treated with Lnc-DIF over-expression plasmid (mean ± SD, *P<0.05, **P<0.01, N=3). ALP: results of Alp staining. ARS: results of Alizarin red staining.

B. Relative staining areas of ALP and Alizarin red staining of MC3T3-E1 cell treated with siRNA-Lnc-DIF (mean ± SD, **P<0.01, ***P<0.001, N=3).

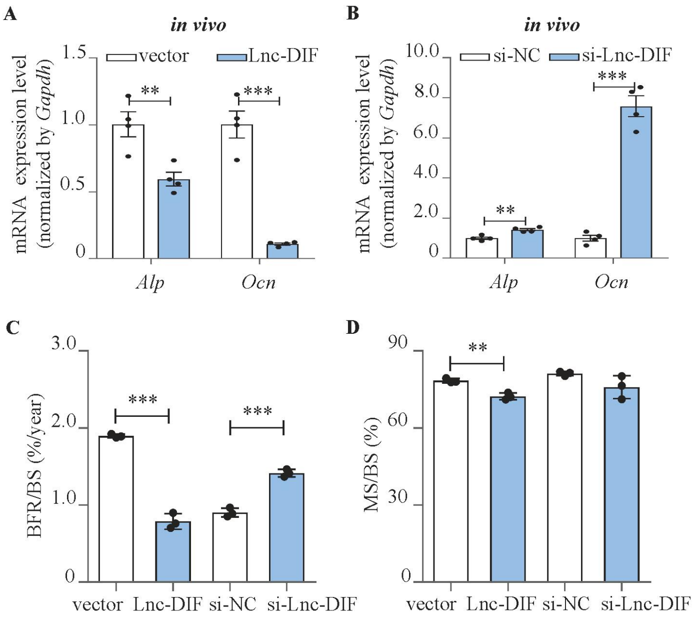

Figure S5. Lnc-DIF inhibited osteoblast differentiation and bone formation *in vivo*. Related to Figure 2.

A. *Alp* and *Ocn* expression levels of C57BL/6 mice BMSCs treated with Lnc-DIF over-expression plasmid, as detected by RT-PCR (mean ± SD, $**P<0.01$, $***P<0.001$, N=4).

B. *Alp* and *Ocn* expression levels of 24 month aging C57BL/6 mice BMSCs treated with siRNA-Lnc-DIF, as detected by RT-PCR (mean ± SD, $**P<0.01$, $***P<0.001$, N=4).

C. Bone formation rate of C57BL/6 mice treated with Lnc-DIF over-expression plasmid or siRNA-Lnc-DIF (mean ± SD, $***P<0.001$, N=3).

D. Mineralizing surface of C57BL/6 mice treated with Lnc-DIF over-expression plasmid or siRNA-Lnc-DIF (mean ± SD, $**P<0.01$, N=3).

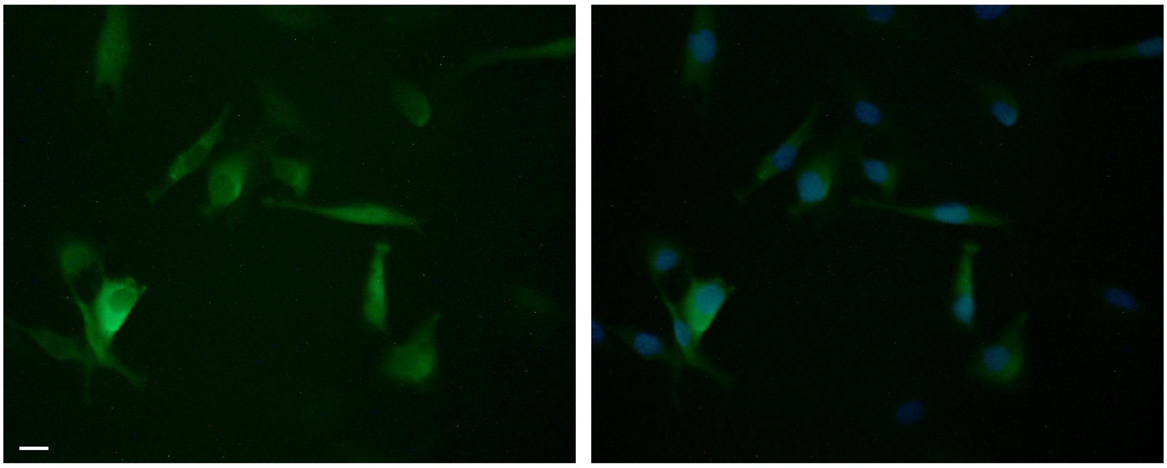

Figure S6. Distribution of Lnc-DIF in MC3T3-E1 cells, as detected by Fluorescence in situ hybridization (FISH). Scale bar: 20μm. Related to Figure 3.

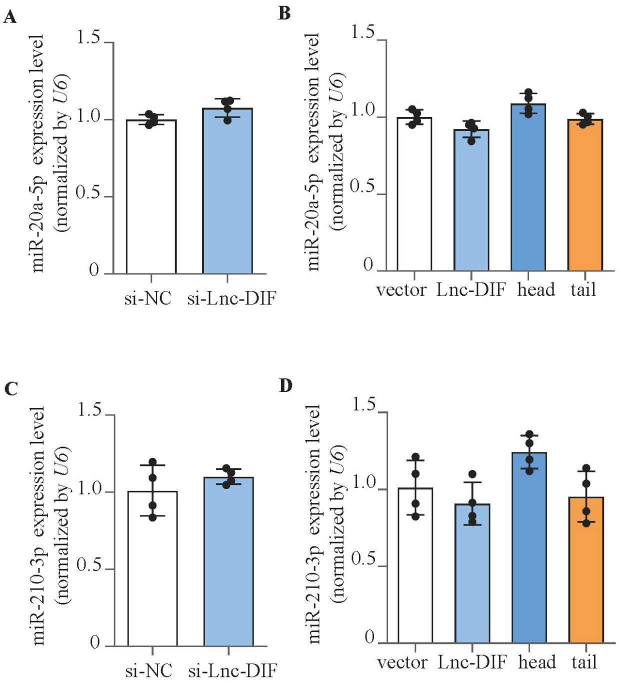

Figure S7. Lnc-DIF did not affect miR-20a-5p and miR-210-3p. Related to Figure 3.

A. miR-20a-5p expression levels of MC3T3-E1 cells treated with Lnc-DIF siRNA, as detected by RT-PCR (mean ± SD, N=4).

B. miR-20a-5p expression levels of MC3T3-E1 cells treated with Lnc-DIF and Lnc-DIF region expression plasmids, as detected by RT-PCR (mean ± SD, N=4). Vector: empty expression plasmid. Lnc-DIF: expression plasmid containing Lnc-DIF full length. Head: expression plasmid containing Lnc-DIF head region. Tail: expression plasmid containing Lnc-DIF tail region.

C. miR-210-3p expression levels of MC3T3-E1 cells treated with Lnc-DIF siRNA, as detected by RT-PCR (mean ± SD, N=4).

D. miR-210-3p expression levels of MC3T3-E1 cells treated with Lnc-DIF and Lnc-DIF region expression plasmids, as detected by RT-PCR (mean ± SD, N=4). Vector: empty expression plasmid. Lnc-DIF: expression plasmid containing Lnc-DIF full length. Head:

expression plasmid containing Lnc-DIF head region. Tail: expression plasmid containing Lnc-DIF tail region.

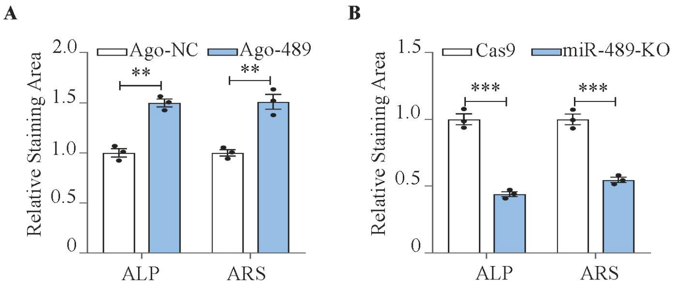

Figure S8. Relative staining areas of osteoblast treated by miR-489-3p. Related to Figure 4.

A. Relative staining areas of ALP and Alizarin red staining of MC3T3-E1 cell treated with agomiR-489-3p (mean ± SD, **P<0.01, N=3). ALP: results of Alp staining. ARS: results of Alizarin red staining.

B. Relative staining areas of ALP and Alizarin red staining of miR-489-3p knock out MC3T3-E1 cell (mean ± SD, ***P<0.001, N=3).

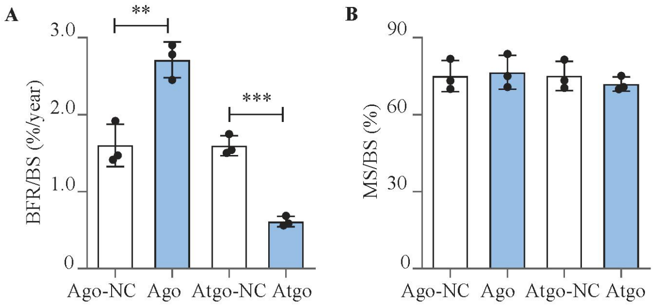

Figure S9. miR-489-3p promoted bone formation. Related to Figure 4.

A. Bone formation rate of C57BL/6 mice treated with agomiR-489-3p or antagomiR-489-3p (mean ± SD, ***P<0.001, N=3). Ago-NC: agomir-NC. Ago: agomiR-489-3p. Atgo-NC: antagomir-NC. Atgo: antagomir-489-3p.

B. Mineralizing surface of C57BL/6 mice treated with agomiR-489-3p or antagomiR-489-3p

(mean ± SD, N=3).

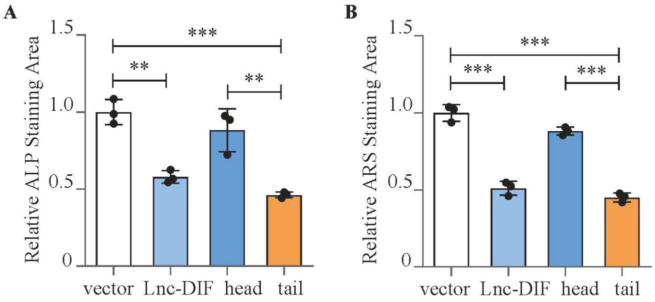

Figure S10. Relative staining areas of osteoblast treated by Lnc-DIF and Lnc-DIF region expression plasmids. Related to Figure 5.

A. Relative staining areas of ALP staining of MC3T3-E1 cell treated with Lnc-DIF and Lnc-DIF region expression plasmids (mean ± SD, ***P*<0.01, ****P*<0.001, N=3). ALP: results of Alp staining. Vector: empty expression plasmid. Lnc-DIF: expression plasmid containing Lnc-DIF full length. Head: expression plasmid containing Lnc-DIF head region. Tail: expression plasmid containing Lnc-DIF tail region.

B. Relative staining areas of Alizarin red staining of MC3T3-E1 cell treated with Lnc-DIF and Lnc-DIF region expression plasmids (mean ± SD, ****P*<0.001, N=3). ARS: results of Alizarin red staining.

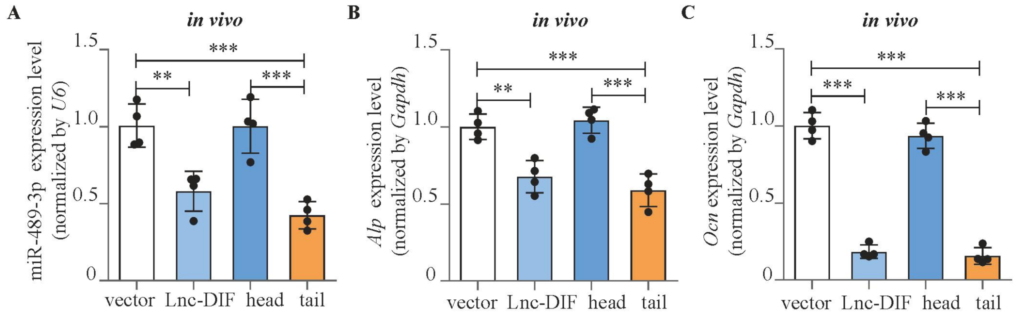

Figure S11. Lnc-DIF region inhibited osteoblast differentiation *in vivo*. Related to Figure 5.

A. miR-489-3p expression levels of C57BL/6 mice BMSCs treated with Lnc-DIF and Lnc-DIF region expression plasmids, as detected by RT-PCR (mean ± SD, ***P*<0.01, ****P*<0.001, N=4). Vector: empty expression plasmid. Lnc-DIF: expression plasmid containing Lnc-DIF full length. Head: expression plasmid containing Lnc-DIF head region. Tail: expression plasmid containing Lnc-DIF tail region.

B. *Col-1* expression levels of C57BL/6 mice BMSCs treated with Lnc-DIF and Lnc-DIF region expression plasmids, as detected by RT-PCR (mean ± SD, ***P*<0.01, ****P*<0.001, N=4).

C. *Runx2* expression levels of C57BL/6 mice BMSCs treated with Lnc-DIF and Lnc-DIF region expression plasmids, as detected by RT-PCR (mean ± SD, ****P*<0.001, N=4).

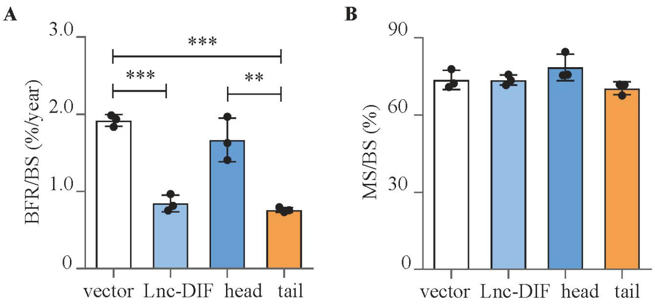

Figure S12. Lnc-DIF region inhibited bone formation. Related to Figure 5.

A. Bone formation rate of C57BL/6 mice treated with Lnc-DIF and Lnc-DIF region expression plasmids (mean ± SD, ***P*<0.01, ****P*<0.001, N=3). Vector: empty expression plasmid. Lnc-DIF: expression plasmid containing Lnc-DIF full length. Head: expression plasmid containing Lnc-DIF head region. Tail: expression plasmid containing Lnc-DIF tail region.

B. Mineralizing surface of C57BL/6 mice treated with Lnc-DIF and Lnc-DIF region expression plasmids (mean ± SD, N=3).

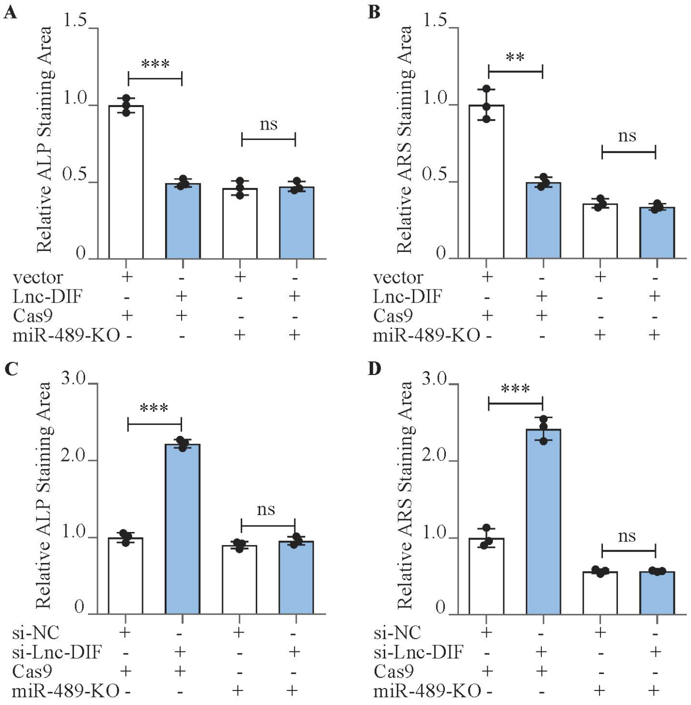

Figure S13. Relative staining areas of miR-489-3p knock out MC3T3-E1 cell treated with Lnc-DIF. Related to Figure 5.

A. Relative staining areas of ALP staining of miR-489-3p knock out MC3T3-E1 cell treated with Lnc-DIF over-expression plasmids (mean ± SD, ***P<0.001, N=3). ALP: results of Alp staining. Vector: empty expression plasmid. Lnc-DIF: expression plasmid containing Lnc-DIF full length.

B. Relative staining areas of Alizarin red staining of miR-489-3p knock out MC3T3-E1 cell treated with Lnc-DIF over-expression plasmids (mean ± SD, **P<0.01, N=3). ARS: results of Alizarin red staining.

C. Relative staining areas of ALP staining of miR-489-3p knock out MC3T3-E1 cell treated with siRNA-Lnc-DIF (mean ± SD, ***P<0.001, N=3).

D. Relative staining areas of Alizarin red staining of miR-489-3p knock out MC3T3-E1 cell treated with siRNA-Lnc-DIF (mean ± SD, ***P<0.001, N=3).

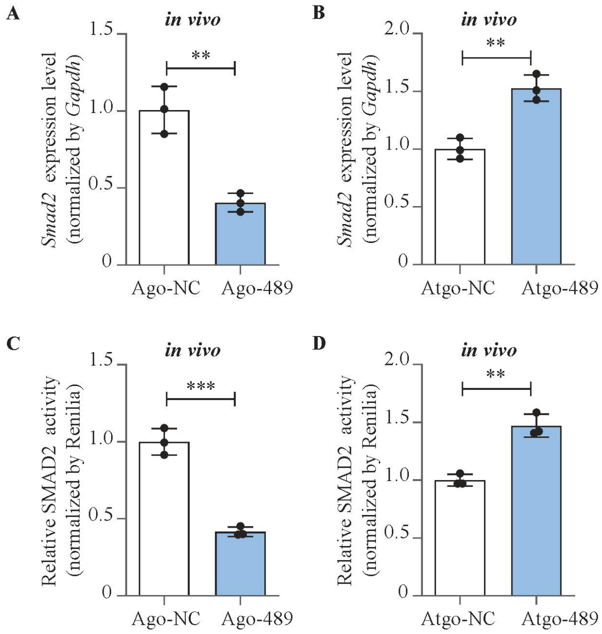

Figure S14. Regulatory effect of miR-489-3p on SMAD2 *in vivo*. Related to Figure 6.

A-B. Effect of miR-489-3p on *Smad2* expression in C57BL/6 mice BMSCs treated with agomiR-489-3p and antagomiR-489-3p, as detected by RT-PCR (mean ± SD, $^{**}P<0.01$, N=3).

C-D. Effect of miR-489-3p on SMAD2 activity in C57BL/6 mice BMSCs treated with agomiR-489-3p and antagomiR-489-3p, as detected by luciferase reporter assay (mean ± SD, $^{**}P<0.01$, $^{***}P<0.001$, N=3).

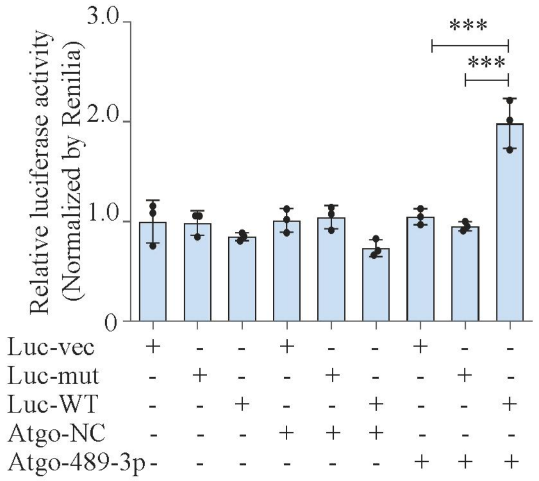

Figure S15. Binding effect of miR-489-3p and *Smad2*-3'UTR, as detected by luciferase reporter assay and treated by antagomiR-489-3p (mean ± SD, ****P*<0.001, N=3). Luc-vec: empty luciferase reporter plasmid. Luc-mut: luciferase reporter plasmid containing mutant *Smad2* 3′UTR. Luc-WT: luciferase reporter plasmid containing wild-type *Smad2* 3′UTR. Related to Figure 6.

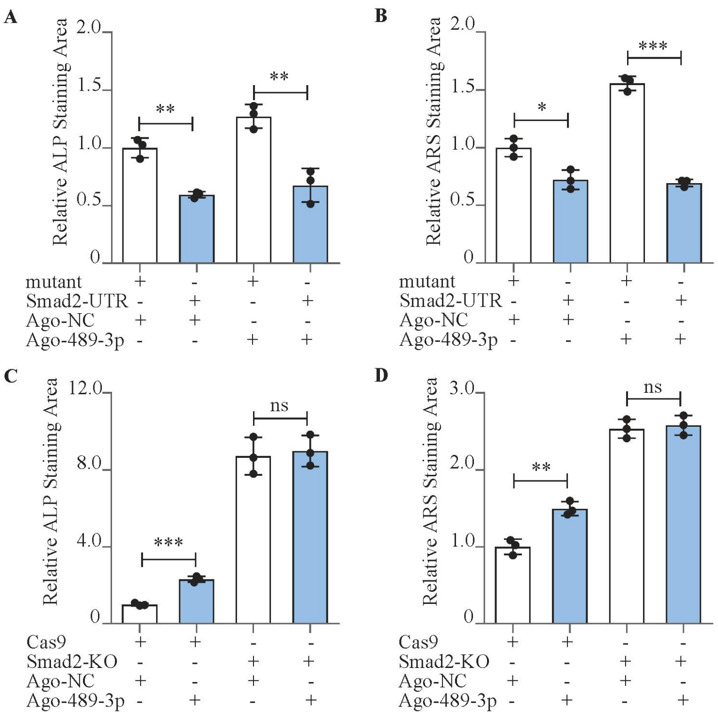

Figure S16. Relative staining areas of MC3T3-E1 cell treated with *Smad2* and miR-489-3p. Related to Figure 6.

A. Relative staining areas of ALP staining of MC3T3-E1 cell treated with *Smad2* 3′UTR plasmid and agomiR-489-3p. ALP: results of Alp staining. Mutant: expression plasmid containing mutant miR-489-3p-*Smad2* 3′UTR binding site sequence. *Smad2*-UTR: expression plasmid containing wild-type miR-489-3p-*Smad2* 3′UTR binding site sequence (mean ± SD, ***P*<0.01, N=3).

B. Relative staining areas of Alizarin red staining of MC3T3-E1 cell treated with *Smad2* 3′UTR plasmid and agomiR-489-3p. ARS: results of Alizarin red staining (mean ± SD, **P*<0.05, ****P*<0.001, N=3).

C. Relative staining areas of ALP staining of SMAD2 knock out MC3T3-E1 cell treated with agomiR-489 (mean ± SD, ****P*<0.001, N=3).

D. Relative staining areas of Alizarin red staining of SMAD2 knock out MC3T3-E1 cell

treated with agomiR-489 (mean ± SD, $**P<0.01$, N=3).

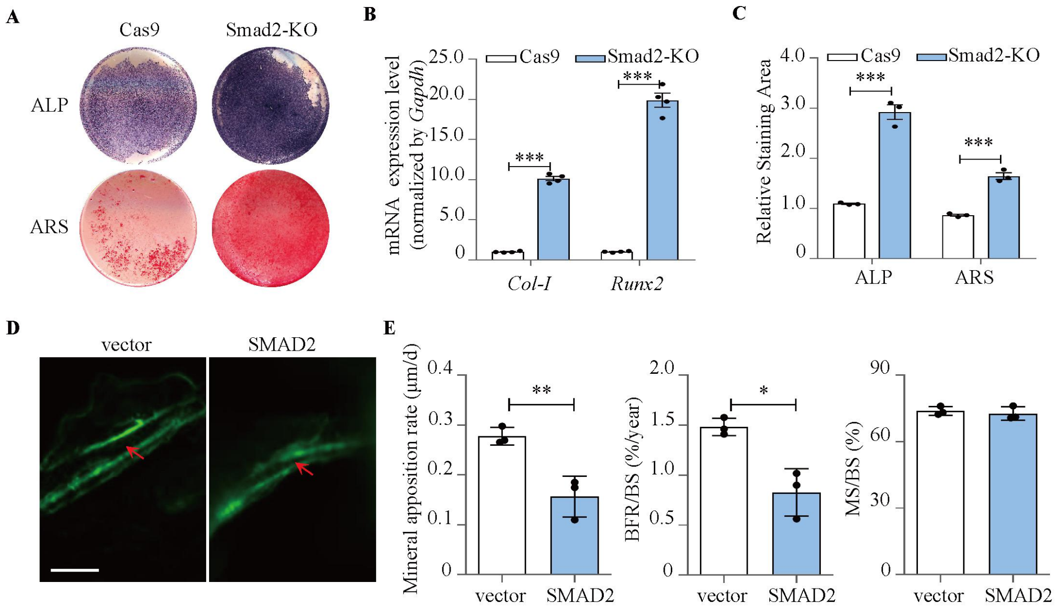

Figure S17. SMAD2 inhibited osteoblast differentiation and bone formation. Related to Figure 6.

A. Alp and Alizarin red staining of SMAD2 knock out MC3T3-E1 cell (compared with blank CRISPR-Cas9 treated MC3T3-E1 cell), as detected by Alp staining and Alizarin red staining. Alp: results of Alp staining. Alz: results of Alizarin red staining.

B. *Col-I* and *Runx2* expression levels of SMAD2 knock out MC3T3-E1 cell, as detected by RT-PCR (mean ± SD, ***P<0.001, N=4).

C. Relative staining areas of ALP and Alizarin red staining of MC3T3-E1 cell treated with SMAD2 overexpression plasmid. Vector: empty expression plasmid. SMAD2: expression plasmid containing SMAD2 (mean ± SD, ***P<0.001, N=3).

D. Representative images showing femoral trabecular bone mineral apposition rate of C57BL/6 mice treated with SMAD2 overexpression plasmid. Scale bar: 10μm.

E. Mineral apposition rate, bone formation rate, and mineralizing surface of C57BL/6 mice treated with SMAD2 overexpression plasmid (mean ± SD, *P<0.05, **P<0.01, N=3).

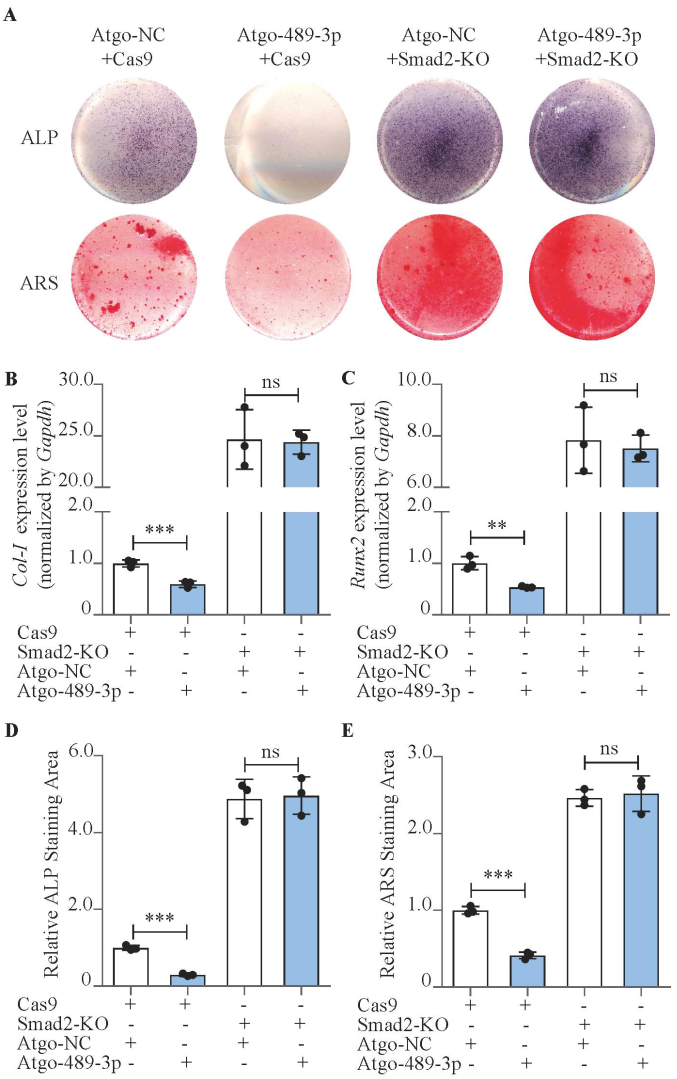

Figure S18. Osteoblast differentiation of SMAD2 knock out MC3T3-E1 cell treated with antagomiR-489. Related to Figure 6.

A. Alp and Alizarin red staining of SMAD2 knock out MC3T3-E1 cell treated with antagomiR-489, as detected by Alp staining and Alizarin red staining. Alp: results of Alp staining. Alz: results of Alizarin red staining.

B. *Col-I* expression levels of SMAD2 knock out MC3T3-E1 cell treated with antagomiR-489, as detected by RT-PCR (mean ± SD, ***P<0.001, N=3).

C. *Runx2* expression levels of SMAD2 knock out MC3T3-E1 cell treated with antagomiR-489, as detected by RT-PCR (mean ± SD, **P<0.01, N=3).

D-E. Relative staining areas of ALP and Alizarin red staining of SMAD2 knock out MC3T3-E1 cell treated with antagomiR-489 (mean ± SD, ***P<0.001, N=3).

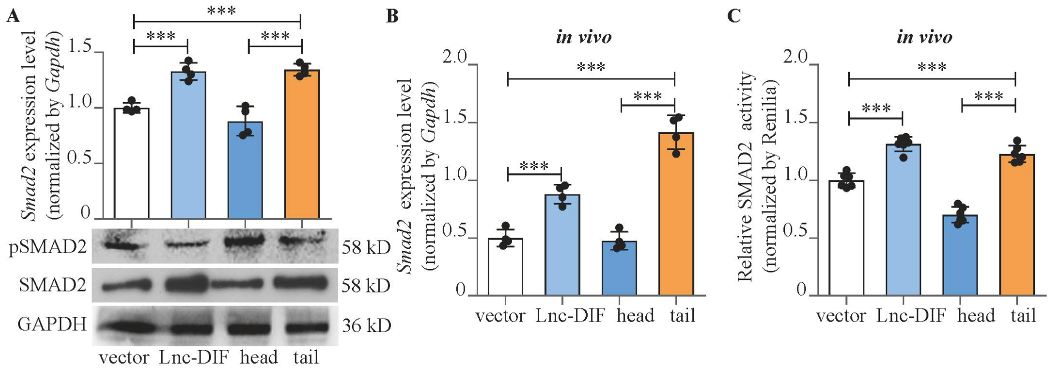

Figure S19. Lnc-DIF region regulated SMAD2. Related to Figure 7.

A. SMAD2 and phosphorylated SMAD2 levels of MC3T3-E1 cells treated with Lnc-DIF and Lnc-DIF region expression plasmids, as detected by RT-PCR and western blot (mean ± SD, ***P<0.001, N=4). Vector: empty expression plasmid. Lnc-DIF: expression plasmid containing Lnc-DIF full length. Head: expression plasmid containing Lnc-DIF head region. Tail: expression plasmid containing Lnc-DIF tail region.

B. *Smad2* expression levels of C57BL/6 mice BMSCs treated with Lnc-DIF and Lnc-DIF region expression plasmids, as detected by RT-PCR (mean ± SD, ***P<0.001, N=4).

C. SMAD2 activities of C57BL/6 mice BMSCs treated with Lnc-DIF and Lnc-DIF region expression plasmids, as detected by RT-PCR (mean ± SD, ***P<0.001, N=6).

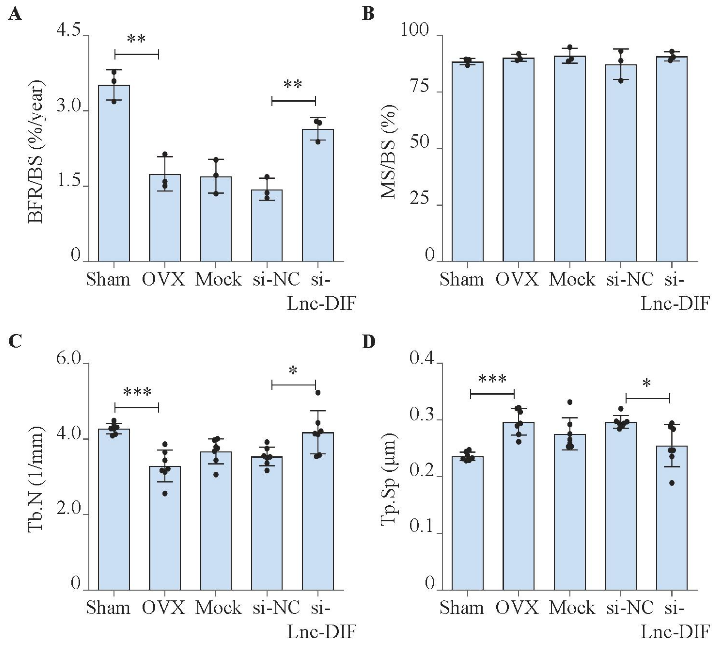

Figure S20. Rescue effect of Lnc-DIF siRNA on trabecular bone microarchitecture and cortical bone formation in OVX mice. Related to Figure 8.

A. Cortical bone formation rate of C57BL/6 mice after OVX treatment and siRNA-Lnc-DIF treatment (mean ± SD, ***P*<0.01, N=3). Sham: sham OVX operation group. OVX: OVX group. Mock: osteoblast-targeting delivery system control group. si-NC: negative control si-RNA treated group. si-Lnc-DIF: siRNA-Lnc-DIF treated group.

B. Cortical mineralizing surface of C57BL/6 mice after OVX treatment and siRNA-Lnc-DIF treatment (mean ± SD, ***P*<0.01, N=3).

C. Trabecular number (Tb.N.) of C57BL/6 mice after OVX treatment and siRNA-Lnc-DIF treatment, as detected by micro CT (mean ± SD, **P*<0.05, ****P*<0.001, N=7).

D. Trabecular separation (Tb.Sp.) of C57BL/6 mice after OVX treatment and siRNA-Lnc-DIF treatment, as detected by micro CT (mean ± SD, **P*<0.05, ****P*<0.001, N=7).

Figure 6A

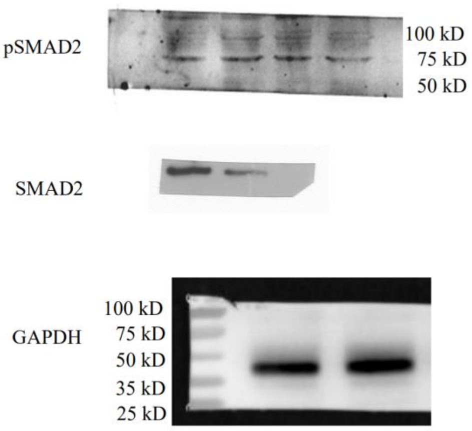

Figure 6B

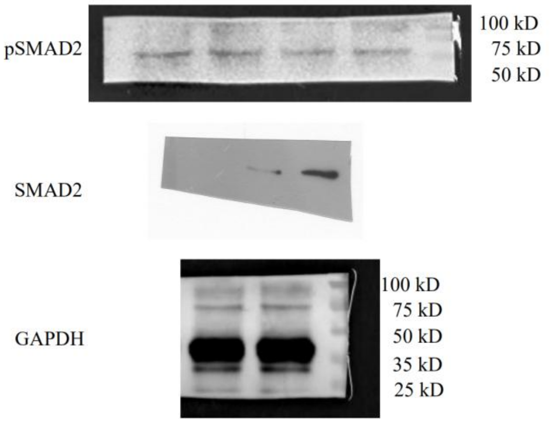

Figure 7B

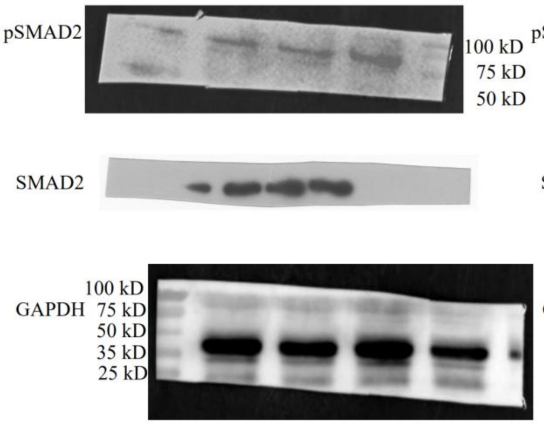

Figure 7C

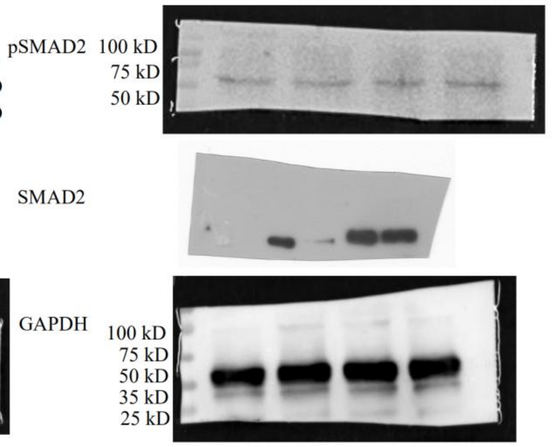

Figure S19A

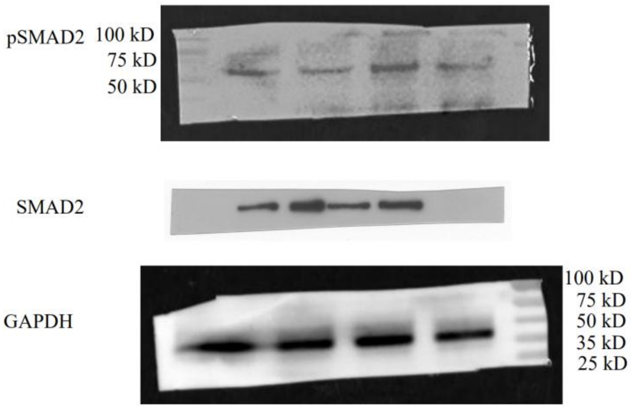

Figure S21. Original images of western blot. Related to STAR Methods.
